# Sculpting and fusing biomimetic vesicle networks using optical tweezers

**DOI:** 10.1038/s41467-018-04282-w

**Published:** 2018-05-14

**Authors:** Guido Bolognesi, Mark S. Friddin, Ali Salehi-Reyhani, Nathan E. Barlow, Nicholas J. Brooks, Oscar Ces, Yuval Elani

**Affiliations:** 1 Department of Chemical Engineering, Loughborough University, Loughborough, LE11 3TU UK; 2 Department of Chemistry, Imperial College London, Exhibition Road, London, SW7 2AZ UK; 3 Institute of Chemical Biology, Imperial College London, Exhibition Road, London, SW7 2AZ UK; 4 FABRICELL, Imperial College London, Exhibition Road, London, SW7 2AZ UK

## Abstract

Constructing higher-order vesicle assemblies has discipline-spanning potential from responsive soft-matter materials to artificial cell networks in synthetic biology. This potential is ultimately derived from the ability to compartmentalise and order chemical species in space. To unlock such applications, spatial organisation of vesicles in relation to one another must be controlled, and techniques to deliver cargo to compartments developed. Herein, we use optical tweezers to assemble, reconfigure and dismantle networks of cell-sized vesicles that, in different experimental scenarios, we engineer to exhibit several interesting properties. Vesicles are connected through double-bilayer junctions formed via electrostatically controlled adhesion. Chemically distinct vesicles are linked across length scales, from several nanometres to hundreds of micrometres, by axon-like tethers. In the former regime, patterning membranes with proteins and nanoparticles facilitates material exchange between compartments and enables laser-triggered vesicle merging. This allows us to mix and dilute content, and to initiate protein expression by delivering biomolecular reaction components.

## Abstract (web summary)

Assembly of higher-order artificial vesicles can unlock new applications. Here, the authors use optical tweezers to construct user-defined 2D and 3D architectures of chemically distinct vesicles and demonstrate inter-vesicle communication and light-enabled compartment merging.

## Introduction

Vesicles are lipid bilayer-encased aqueous compartments that range from attoliters to picoliters in volume. They are widely used as model architectures in studying the biophysical properties of membranes^1^, as functional units in biotechnology (e.g., for applications in bio-sensing^2^, drug delivery^3,4^ and diagnostics^5,6^), as miniaturised reaction vessels^7–9^, and as soft-matter microsystems^10^. These developments have led to increasing interest in using cell-sized giant vesicles as plasma membrane mimics in bottom-up synthetic biology, where they act as a chassis for artificial cells that contain biomolecular components and perform cell-mimetic functions^11–14^. The key feature at the heart of these applications is compartmentalisation: isolation of encapsulated cargo from the external environment, allowing ordering of chemical species in space. In principle, there is a second degree of ordering that can take place, which defines the spatial organisation of individual compartments in relation to one another. In biological systems, this manifests itself in the form of tissues, where cells connect with adjacent cells to order them in space. This, together with intercellular communication, enables cells to exhibit higher-order behaviours as a collective. Mimicking this in vesicle-based systems is likewise expected to result in a step change in utility in a suite of applications, particularly if molecular cargo can also be delivered to vesicle interiors on demand.

Several platforms have been previously developed that involve forming higher-order networks of cell-like compartments using related but fundamentally different structures to vesicles. Water-in-oil droplet interface bilayer (DIB) networks have been assembled and engineered to exhibit collective properties, such as self-folding and selective transmission of signals down defined neural-like paths^15,16^, as well as being functionalized with biomolecules to act as simple electrical devices^17,18^.

Other systems involve single vesicles that have been divided into sub-compartments linked by open tethers that are pulled using a micropipette^19–21^, or by pre-assembling internal bilayer partitions^22^. In these structures, however, compartments can still be considered as part of a single vesicular structure: they are encased by a single continuous membrane, and in the former example there is no separation of content between their interiors. These structures are more akin to extended lipid bodies found in organelles such as the endoplasmic reticulum, rather than isolated cells coupled through junctions. In addition, they are not formed by linking pre-existing differentiated vesicles from a population and networks were being confined to 2-D.

There are also impressive examples employing principles of self-assembly to construct vesicle aggregates by embedding molecular recognition modules in the membrane^23,24^, including complementary DNA strands^25,26^. Critically, as they are bulk assemblies, networks of defined architectures cannot be formed, and they are instead colloidal aggregates.

Herein, we develop a different approach that allows us to sculpt and manipulate vesicle networks with fine spatiotemporal control. We engineer an in vitro system composed of adherent cell-sized vesicles coupled to an optical tweezer setup, which enables us to selectively connect isolated vesicles from different sub-populations together to generate networks of user-defined architectures. We term these double bilayer delineations as vesicle interface membranes (VIMs).

Our networks are reconfigurable, can be assembled/disassembled on demand and can have their morphology modulated by external stimuli such as temperature and chemical concentration. Chemically distinct compartments could be physically linked over small (several nm) and large (100s µm) distances by closed tethers. Patterning membranes with proteins and nanoparticles facilitate inter-compartment communication across the intermembrane space between adhered vesicles and allows vesicle merging to be triggered using light. This enables us to perform discrete operations on compartments, such mixing and dilution, and to initiate protein expression by delivering transcription/translation biomolecular cargos to a vesicle containing a plasmid. We envisage that this platform could be deployed for the development of new biomaterials, synthetic cell networks, minuturised bioreactors, implanted therapeutic devices and responsive materials.

## Results

### Assembling vesicle networks

**Fig. 1:**
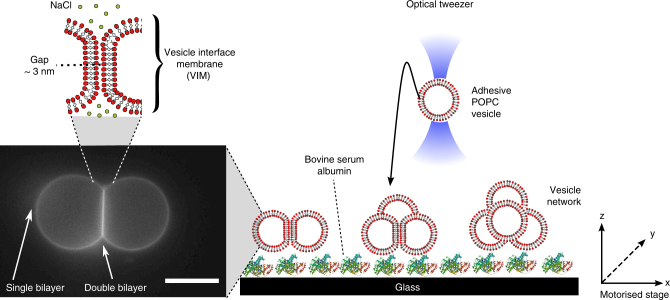
Schematic of setup for the controlled assembly of vesicle networks of user-defined architectures. The presence of 0.75 M NaCl in the external solution yielded adhesive vesicles that could be manipulated in 3-D using an optical tweezer and a motorised stage. System components are not drawn to scale. Vesicle = c. 5 µm radius, laser beam waist = c. 1 µm. Scale bar = 10 µm

Networks were assembled using optical tweezers to deposit vesicles in defined locations, and by exploiting non-specific global intermembrane forces to mediate membrane adhesion. We designed an iso-osmotic system where vesicles made of 1-palmitoyl-2-oleoyl-sn-glycero-3-phosphocholine (POPC) and 1 wt.% 1,2-dioleoyl-sn-glycero-3-phosphoethanolamine-*N*-(lissamine rhodamine B sulfonyl) (ammonium salt) (Rh-PE) could be trapped by optical tweezers (*λ* = 1070 nm) due the refractive index (RI) mismatch between each vesicle’s interior (1.5 M sucrose; RI = 1.406) and exterior (0.75 M NaCl; RI = 1.342) (see Supplementary Notes 1 and 2 for details). Vesicles could be trapped with conditions as low as 0.4 M sucrose (interior) and 0.2 M NaCl (exterior). Depending on the size of the vesicles, we could drag and drop individual vesicles simply by turning the laser on and off, at powers ranging from 80 to 470 mW at the back aperture of the objective (23–190 mW at trap). Networks were assembled by depositing vesicles immediately adjacent to one another (see Fig. 1 for schematic of experimental setup). Seconds after contact, the membranes quickly adhered by zipping up from an initial contact point to form a vesicle pair connected by a membrane patch, termed a VIM (see Supplementary Movie 1).

The formation of an adhesive patch is governed by a balance between attractive Van der Waals forces and repulsive electrostatic, undulation and hydration forces that exist between the two membranes^27^. Although POPC is zwitterionic at neutral pH, it has a slight negative charge^28^, likely due to the orientation of the headgroups^29^ and hydration layers^30^. The presence of NaCl served to screen membrane electrostatic repulsion, allowing the attractive forces to dominate, which resulted in adhesion upon contact. We found that below a critical threshold of 0.2 M NaCl, we could no longer reliably assemble VIMs with well-defined interfaces. As expected, larger NaCl concentrations led to larger VIM interfaces, and the presence of charged lipids increased the minimum concentration of NaCl needed for adhesion (see Supplementary Note 3, Supplementary Fig. 1 and Supplementary Table 1).

**Fig. 2:**
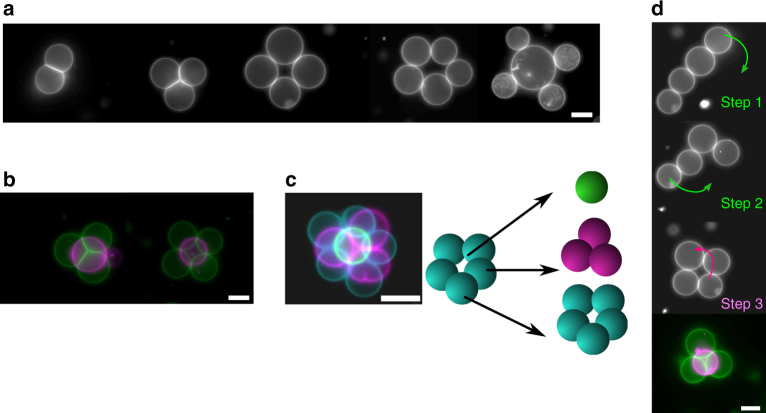
Controlled and reconfigurable assembly of vesicle networks. **a** A defined number of vesicles were brought together to assemble 2-D networks of various architectures, including, from left to right, bi-compartment, trigonal, square, pentagonal and branched geometries. **b** Vesicles were vertically manipulated and deposited enabling 3-D structures to be assembled, including tetrahedral and square-pyramidal geometries. **c** Three-layered pyramid geometries could also be formed, with the vesicle arrangements shown in schematic on the right. **d** Optical tweezers were used to reconfigure the networks between several geometries (linear to square to tetrahedral; arrows indicate direction of movement). False colours used to demonstrate that images were taken in different planes and subsequently superimposed. Green channel = lower plane; pink channel = upper plane. Scale bar = 10 µm for all images. NaCl concentration = 0.75 M

This approach could be used to assemble larger-scale networks of user-defined geometries, simply by dragging and dropping individual vesicles at set locations (Fig. 2a). For example, we were able to form 2-D networks of trigonal, square and pentagonal geometries, as well as branching networks of three vesicles linked to a central node. Our ability to manipulate vesicles in the *z*-direction allowed us to assemble 3-D geometries such as tetrahedrons, square pyramids and three-layered pyramids (Fig. 2b, c). The VIM networks were reconfigurable, in that transformation between geometries was possible (Fig. 2d and Supplementary Movie 2). It was possible to reconfigure a vesicle chain of four vesicles to a 2-D square, and then to a 3-D tetrahedron geometry by lifting one of the vesicles above the remaining three vesicles.

We could controllably transport these networks in space by trapping a single vesicle and dragging the entire adhered network. All the adhered vesicles moved in synchrony, confirming the presence of adhesion patches and showing the VIM networks can be considered discrete assemblies able to be manipulated as a whole.

**Fig. 3:**
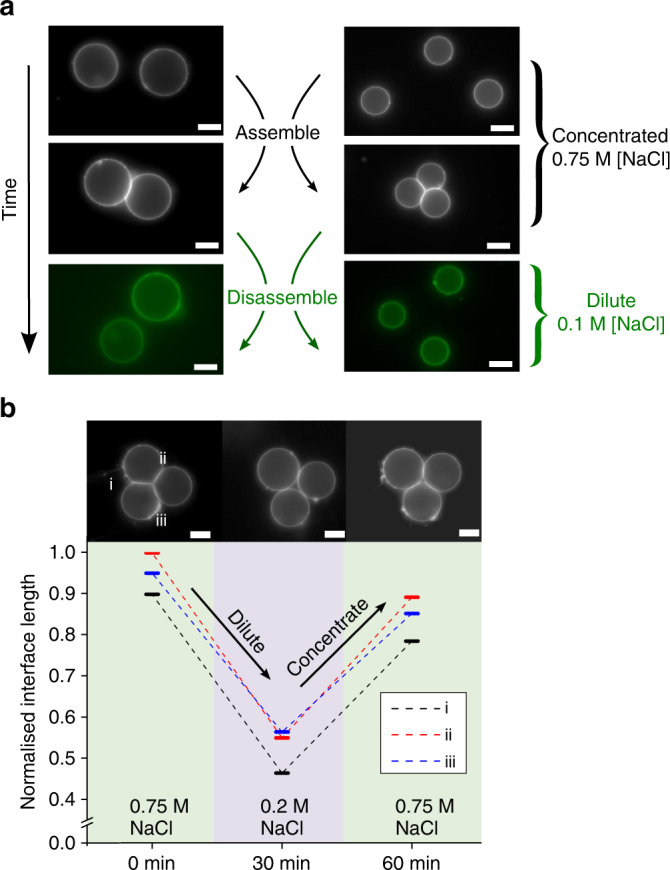
Network modulation using salt concentration. **a** Networks were dismantled simply by reducing NaCl concentration in the exterior by dilution with water. Full detachment occurred after c. 45 min. **b** VIM length was dynamically altered by adjusting NaCl concentration over time, as shown by the relative lengths of the three interfaces in a three-vesicle network. Salt concentration was reduced from 0.75 M to 0.2 M, then increased from 0.2 to 0.75 M after 30 min. Interface length was normalised relative to vesicle radius. Scale bar = 10 µm for all images

Networks could be disassembled after generation by diluting the NaCl concentration below the critical threshold for adhesion (Fig. 3a). This was achieved by slow perfusion of DI water through an agarose gel situated above the sample over a period of minutes, with complete detachment observed after ca. 45 min. Diluting NaCl from 0.75 to 0.2 M also enabled VIM morphology to be modulated, specifically the intermembrane contact angle and the area of the adhesive patch (Fig. 3b). It is worth noting that salt dilution causes an osmotic pressure imbalance across the vesicle membranes and that the resulting increase in membrane tension contributes to the reduction of the intermembrane contact area.

### Vesicle interface membranes characterisation and modulation

**Fig. 4:**
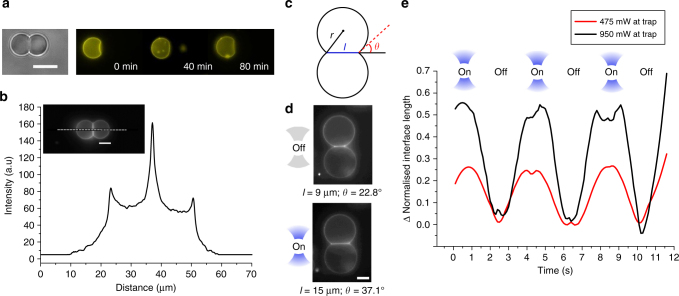
Structural arrangement of VIMs and dependence on temperature. **a** Lack of diffusion of membrane-embedded Rh-PE between vesicle compartments suggests that the vesicle interfaces are composed of two adherent membranes as opposed to a hemifused diaphragm. **b** This is reinforced by a fluorescence intensity profile across a two-vesicle network (inset; dotted line) showing the fluorescence of the interface membrane being double that of the external vesicle membrane. **c** Cartoon showing the characteristic parameters of a symmetric vesicle pair including vesicle radius (*r*), contact angle (*θ*) and interface length (*l*). **d** Turning on the optical tweezers to trap a vesicle (950 mW at trap; bottom vesicle trapped) triggered a change in both interface length and contact angle. **e** Network morphology could be dynamically alternated by sequential application and removal of the trap. This is demonstrated by the graph showing the change in interface length over time as the trap is tuned on and off, where the interface length is normalised with respect to vesicle radius. Lower applied powers resulted in smaller changes to interface lengths. Scale bars = 10 µm for all images

In order to determine whether the VIM was a hemifused structure composed of a single bilayer or a membrane patch consisting of two adhering bilayers, we performed an assay using fluorescently labelled lipids, where one vesicle was labelled with 1 wt.% fluorescent lipid Rh-PE, and the other unlabelled (Fig. 4a). No free diffusion of lipid between vesicles was observed over 80 min, in contrast to hemifused membranes where mixing in the outer bilayer leaflet would be seen within seconds^31^, thus confirming the existence of two distinct adherent membranes^32^. This is further substantiated by obtaining a fluorescence intensity profile across two fluorescently tagged adhered vesicles (Fig. 4b), which shows the adhesion patch having double the intensity of a single bilayer, suggesting the presence of a two bilayer-thick VIM (see Supplementary Note 4 and Supplementary Fig. 2). The VIM membrane was found to be 1.9 times higher in intensity on average than the non-VIM membrane for each vesicle pair (s.d. = 0.17; *n* = 20).

One of the key parameters associated with these structures is the adhesion energy between individual vesicles, which is determined by the summation of the inter-vesicle forces^27^. The net adhesion energy (*W*) can be deduced without accessing the contributions from the individual components simply using the contact angle between membranes of two spheroidal vesicles (*θ*; Fig. 4c) and membrane area expansion modulus (*K*) according to Eq. (1), an approach adopted elsewhere^33^ (see Supplementary Note 5 for details):

1 $${\mathrm{cos}}\,\theta \approx 1 - \left( {\frac{{2W}}{K}} \right)^{\frac{1}{3}}$$

VIM contours and contact angles were extracted using a MATLAB image analysis script (Supplementary Note 6 and Supplementary Fig. 3) and a value for *K* of 213 mJ m^−2^ obtained from literature^34,35^. Adhesion energy at room temperature was determined to be 0.9 mJ m^−2^ (s.d. = 0.3 mJ m^−2^; *n* = 20), similar to what was obtained in other systems^27,35,36^.

The morphology of the vesicle pair could be dynamically tuned by varying the applied laser power, which in turn affects local temperature^37^. As this increases, the membrane area expands, hence alleviating the membrane stress and promoting the expansion of the contact area. However, the temperature change also influences the expansion modulus^38,39^ and the intermolecular forces, which determine the net adhesion energy^25^, making difficult to predict a priori the temperature dependence of the adhesion area. By locally heating the VIM using a laser directed at the centre of one of the vesicles (0.95 W at trap), it was experimentally observed that the interface length increased from 9 to 15 µm and the contact angle increased from 22.8° to 37.1° (Fig. 4d). These values could be modulated by varying the applied laser power (Fig. 4e). We could switch between the morphologies simply by turning the laser source on and off, with conversion between the two arrangements occurring in seconds since the characteristic time for heat dissipation in water is lower than 1 ms at the vesicle length scale (Fig. 4e). The local temperature increase due to the delivered energy from the laser was estimated to be 9 °C (Supplementary Note 8 and Supplementary Table 2). Similar results were seen when the vesicle was heated using a heating stage and not with a laser, further suggesting that this is a temperature-mediated effect (Supplementary Fig. 5A).

It is worth noting that the contact area between the two similarly sized vesicles remained flat during the modulation, despite the fact that the trap was centred on one of the vesicles, and not symmetrically located between the two vesicles. This suggests that no mismatch in the vesicle mechanical properties was induced by the optical tweezer, hence ruling out any optical stretching effect on the equilibrium VIM morphology. Furthermore, a similar modulation of VIM morphology, although within a narrower range, could also be achieved when the trapping beam was located a few microns distant from the vesicles, the latter no longer being optically trapped (Supplementary Note 8 and Supplementary Fig. 5B).

A second key parameter to deduce is the distance between the adherent membranes. This was acquired by obtaining small-angle X-ray scattering (SAXS) of POPC multi-lamellar stacks with 0.75 M NaCl (Supplementary Fig. 6), revealing a d-spacing 6.9 nm. Together with a value for POPC bilayer thickness of 3.8 nm (2_zP_, distance between the PC headgroups) obtained from literature^40^, an intermembrane distance of 3.1 nm was calculated. These values will not be identical in adhered vesicles but will likely be comparable (Supplementary Note 9). In adhered vesicles, undulations are dampened due to membrane stretching, which may also lead to hydrophobic attractions in the bilayers due to the exposed hydrophobic core^27^. In addition, POPC bilayers are estimated to thicken by up to 2 Å in the presence of salt^41^, which would further reduce the estimated intermembrane distance. The intermembrane value obtained is therefore likely to be an upper-bound value, as it ignores these considerations.

### Connection over large distances through lipid tethers

In addition to linking compartments immediately adjacent (several nm) to one another, we were also able to form tethers that physically connect two chemically distinct vesicles across large distances in space (hundreds of microns). There have been previous examples of controllable tether formation between beads and vesicles^42,43^ using traps, but here we form tethers between multiple vesicles.

**Fig. 5:**
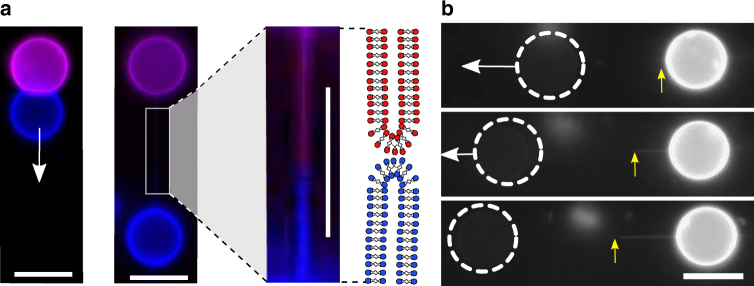
Linking vesicles over long distances through tethers. **a** Tethers between two vesicles were formed when one vesicle was pulled relative to another stationary surface-adhered vesicle. White arrow indicates direction of pulling. Labelling each vesicle with a different fluorophore (Rh-PE = purple; NBD-PE = blue) demonstrates the existence of two sub-tethers, one from each vesicle, which met at the middle and anchored on a sub-micron adhesion patch. **b** The precise location of the anchor point (yellow arrow) could be varied by extending the tether by pulling the vesicle. Dotted white circles represent location of a non-fluorescence vesicle. Scale bar = 10 µm for all images

Once formed, if VIMs were left to sediment on a coverslip substrate which was not passivated with bovine serum albumin (BSA), stochastic adhesion of a vesicle on the surface could be observed. The second non-adhered vesicle could thus be moved using optical tweezers, while the partner remained stationary. Surprisingly, during this process, complete fission of the adhesion patch was not observed. Instead, as the vesicle was pulled the adhesion area was found to decrease until a certain point where an interconnecting tether appeared between the two vesicles (Fig. 5, Supplementary Note 7 and Supplementary Fig. 4).

This tether could be pulled for hundreds of microns without breaking. Tether rupture was never observed and increasing the speed at which the tether was pulled led to the vesicle escaping the optical tweezer, demonstrating that optical forces never exceed the tether-tether adhesion force or the tether rupture force. Upon release of the vesicle by turning off the trap, the tether retracted and the VIM reformed an adhesion patch, a process that could be repeated multiple times. These tethers were closed, as demonstrated by lack of diffusion of encapsulated calcein and of membrane-embedded Rh-PE fluorescent lipids between compartments over 20 min (Supplementary Note 10 and Supplementary Fig. 7). This suggests that there is a minute adhesive patch holding the tether together, similar to those found when tethers are formed with streptavidin-labelled beads and biotinylated vesicles^43^.

Labelling one vesicle with fluorescent lipid enabled us to locate the anchor point between the membranes. This led to the striking observation that in most cases the tether was not composed of a membrane from only one vesicle, but indeed was composed of both, with membranes meeting near the tether midpoint (Fig. 5a and Supplementary Movie 3). On occasions when the anchor point was asymmetrically positioned along the tether (i.e., lying closer to one vesicle), pulling the tether farther led to movement of the anchor point until it reached the midpoint (Fig. 5b).

Uniquely, the tethers we formed are held together with non-specific global forces as opposed to specific molecular forces such as biotin/streptavidin or DNA base pair interactions. One hypothesis is that the formation of tethers is governed by a trade-off between an energetic penalty in breaking the membrane patch and that of deforming the membrane area. Initially, at a large VIM adhesion patch, there is a large energetic cost to pull a tether as the deformation can be resisted by surface tension. The patch therefore decreases in size until a point where it is small enough so that the energetic cost in pulling the tether is lower than breaking the remaining adhesion patch.

### Vesicle communication

One of the grand promises of compartmentalised vesicle soft-matter assemblies is the prospect of using them as picolitre/femtolitre reaction vessels, and as tissue-like cell-mimetic compartments in synthetic biology. To realise this, communication and material transfer between compartments must be demonstrated. This poses a challenge in the VIM system, as the presence of a double membrane at the interfaces precludes the use of single transmembrane protein pores as conduits between compartments.

To tackle this, we use the protein pore alpha-Hemolysin (α-HL; pore diameter 1.4 nm), and rely on diffusion through two pores, one to allow material to leave the donor vesicle into the intermembrane space, and a second to allow it to diffuses from this space to the acceptor vesicle. However, as α-HL inserts into all membranes—both those at the vesicle interface and those facing the external solution—we had to selectively block channels not lying in the intermembrane region to prevent leakage of cargo into the external environment. This was achieved by adding the cyclodextrin blocker (heptakis(2,3,6-tri-O-methyl)-β-cyclodextrin; TRIMEB) to the external solution, which non-covalently occludes the α-HL pore^44^ thus reducing leakage of material to the exterior^45,46^. Crucially, as the blocker is slightly larger than the intermembrane distance (maximum point to point distance ca. 4 nm)^47^, its ability to penetrate this space is diminished, allowing selective translocation of material between compartments. Although α-HL can diffuse out of the VIM area, when it does so, it encounters blockers present in the external solution, inhibiting leakage of encapsulated material.

**Fig. 6:**
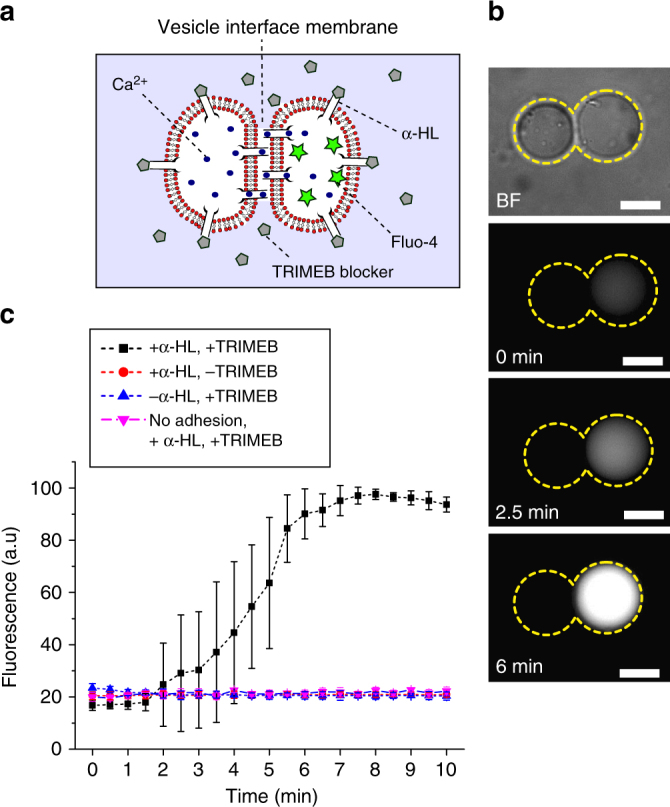
Inter-vesicle communication. **a** Schematic of the engineered system enabling encapsulated content (Ca^2+^) to translocate selectively through two sequential protein pores, into the adjacent compartment containing a Ca^2+^ sensitive dye (Fluo-4). Leakage of ions through the external membranes is suppressed by the presence of channel blockers, which have a diminished ability to penetrate into the intermembrane space. **b** Brightfield and fluorescence images of an adhered vesicle pair showing successful communication, with the dye-containing vesicle increasing in fluorescence over time. Dotted yellow lines represent the vesicle boundaries. **c** Graph of fluorescence intensity in the dye-containing compartment over time. Error bars represent standard deviation (*n* = 5). Scale bar = 10 µm for all images

To confirm successful operation of this system, we conducted a fluorescence leakage assay (Fig. 6) between two vesicles containing α-HL (50 ng µl^−1^) internally, formed via the emulsion transfer method. A donor vesicle was loaded with Ca^2+^ (200 mM), an acceptor vesicle with the Ca^2+^ sensitive Fluo-4 dye (0.54 mM) and EDTA (1 mM) to minimise background fluorescence, with TRIMEB (10 mM) in the exterior (see Fig. 6a for schematic). After a variable lag phase of up to 4 min (Supplementary Note 11 and Supplementary Fig. 8), the acceptor compartment increased in fluorescence as Ca^2+^ diffused through the pores, until the signal saturated after ca. 5 min (Fig. 6b, c). No fluorescence increase was seen in the control measurements with no protein present or with no blocker present due to leakage of Ca^2+^ to the exterior. Likewise, fluorescence did not increase when NaCl was absent in the exterior, but α-HL and TRIMEB were present internally. Vesicles were brought into contact, but no VIM formed. This result indicates that where successful inter-vesicle communication was seen, Ca^2+^ was not diffusing into the bulk and then back into the adjacent vesicle. We also ran fluorescence release experiments on single vesicles to demonstrate the effectiveness of the blocker at preventing ion leakage into the external environment, and results showed that this indeed slowed the rate by fivefold (Supplementary Note 12 and Supplementary Fig. 9). It should be noted that there is likely a residual level of leakiness in the system due to material escaping through the intermembrane space and, due to imperfect blocking of the pores, through the external membranes.

### Vesicle fusion

**Fig. 7:**
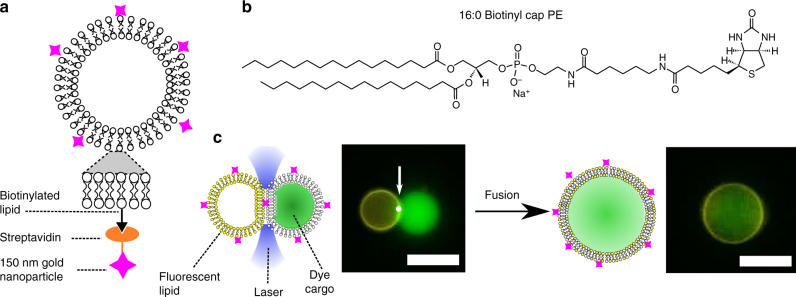
AuNP-mediated vesicle fusion. **a** Schematic of the AuNP-labelled vesicle. **b** Chemical structure of the biotinylated lipid. **c** Schematic and fluorescence microscopy image of VIM fusion. A Rh-PE-labelled vesicle (yellow channel) is fused with a calcein-containing vesicle (green channel) with the application of a laser (white dot) at the VIM (white arrow) to yield a vesicle carrying both fluorescent lipid and fluorescent cargo. Scale bar = 5 µm for all images

**Fig. 8:**
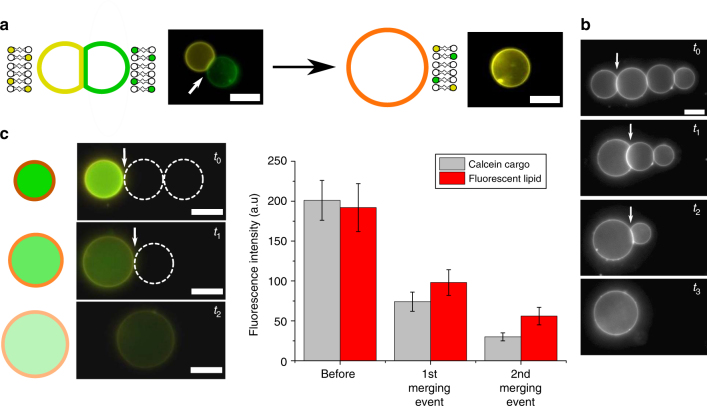
Vesicle network fusion and material mixing. **a** Schematic and fluorescence images of a Rh-PE (yellow) and NBD-PE (green)-labelled vesicle fused to yield a larger vesicle containing both lipids. **b** A four-vesicle network where each vesicle junction is ruptured in turn by the laser, demonstrating spatial control of the process. **c** Dilution of both the cargo (calcein; green) and membrane material (Rh-PE; yellow) of a vesicle through sequential merging with two empty vesicles (dotted white circles). Graph shows mean fluorescence intensity of cargo and membrane (error bars = 1 s.d.; *n* = 10). White arrows show the VIM selected to fuse. Scale bars = 5 µm for all images

By slightly altering our experimental system, it was possible to use the optical tweezer to fuse selected compartments in a VIM network, an operation that can be performed with high temporal and spatial resolution. To do this, we attached 150 nm gold nanoparticles (AuNP) on the outer surface of the vesicles using biotin/streptavidin conjugation (Fig. 7a, b). When these enter the focus of the laser, they absorb energy which is dissipated as heat, leading to an extreme temperature increase (>100 °C using our operating powers)^48^. This is a localised effect with heat dissipation occurring on the length scale comparable to the nanoparticles^48^. The disruption to the membrane fabric (possibly through membrane expansion and opening of a fusion pore)^49^ was enough to lead to breakdown of the VIM and fusion of the compartments at sufficiently high powers (>150 mW at trap; see Supplementary Movie 4)^49,50^. Fusion was complete several seconds after focusing the laser on the VIM. Alternatively, 80 nm AuNPs could also be used, although the powers needed to consistently achieve fusion were higher (>300 mW at trap)^48^. With 1–2 nm AuNP, fusion was not observed, likely due to the short heat dissipation distances and because they do not resonate at the trapping laser wavelength. Adding calcein dye (50 mM) to one vesicle and fluorescently labelled lipid (Rh-PE) to the other showed that both the vesicle lumen and the membrane itself were completely mixed post-fusion (Fig. 7c). The potential to alter the membrane composition of the fused GUV was demonstrated by fusing two GUVs with a different fluorescently tagged lipid each (Rh-PE and NBD-PE; Fig. 8a). The location of the AuNP on the membrane is still unknown. If the particle does indeed sit in the intermembrane space, it has the potential to form a biotin/streptavidin bridge between the vesicles. However, as the addition of AuNPs does not significantly affect the size of the adhesion patch (Supplementary Note 13 and Supplementary Fig. 10) this effect is not thought to be significant.

As the thermally triggered fusion was localised to the laser spot, we were able to construct a VIM network, and specifically select a single discrete VIM to be fused. For example, we formed a four-vesicle network, fusing each one of the VIMs in turn, until only a single large vesicle remained (Fig. 8b). This approach enabled us to perform a sequential dilution both of the lipid membrane material and of the encapsulated material of a cargo-containing vesicle. A vesicle labelled with 1 wt.% fluorescent Rh-PE and encapsulated calcein (50 mM) was fused with two other non-fluorescent vesicles in a VIM network (Fig. 8c). Each fusion event led to a decrease in fluorescence intensity of the encapsulated material and of the membrane of the cargo-containing compartment.

**Fig. 9:**
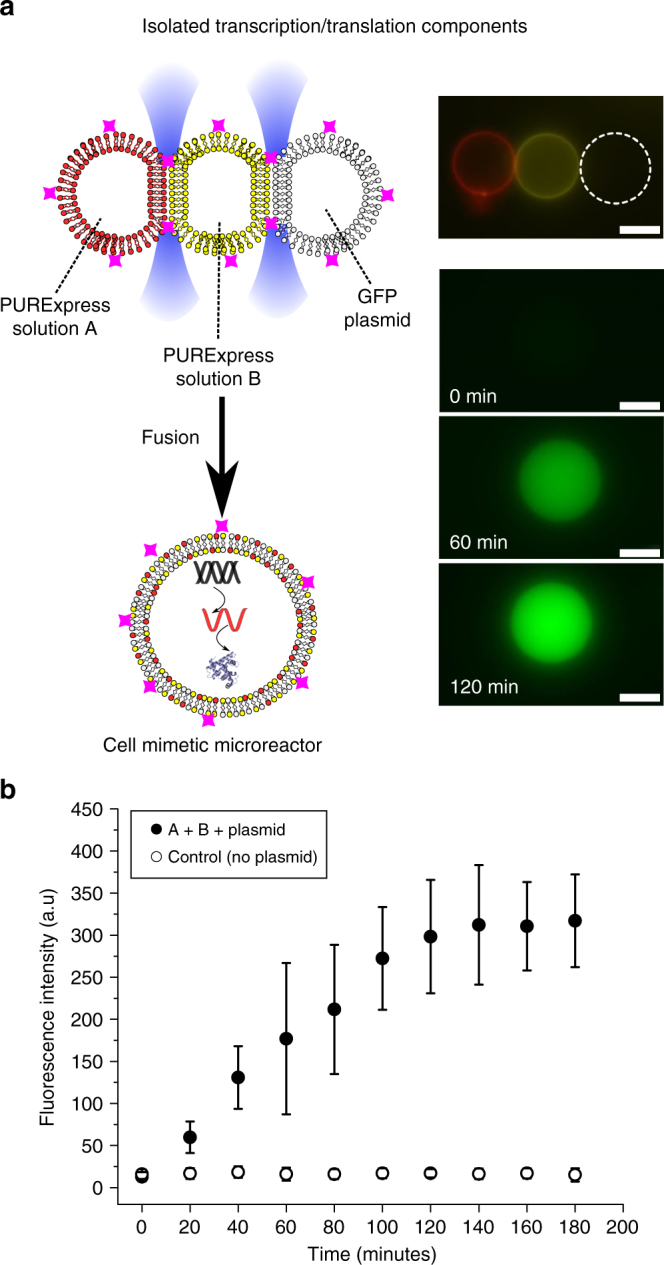
Vesicle fusion to initiate biochemical reactions in a cell-mimetic compartment. **a** Schematic and fluorescence microscopy images of a three-vesicle network containing the different components needed for cell-free protein expression. Red = Cy5-PE lipid, yellow = Rh-PE lipid. Dotted white circle represents unlabelled vesicle. Vesicles were fused with a laser, leading to GFP synthesis (green channel). Scale bar = 5 µm for all images. **b** Graph showing an increase in mean fluorescence following fusion as GFP is being synthesised (error bars = 1 s.d, *n* = 5)

Next, we demonstrated the ability to conduct controlled biochemistry in the vesicles, using them as cell-like reactors. We isolated the components needed for coupled cell-free transcription and translation in three distinct compartments on a VIM network, then initiated protein synthesis using the laser (Fig. 9). We used the PURExpress protein expression system reconstituted from purified cellular components^51^, a GFP plasmid, and a three-vesicle network for these experiments. One vesicle contained tRNAs, amino acids and rNTPs (PURExpress solution A). A second contained ribosomes, T7 RNA polymerase, translation factors, aminoacyl-tRNA synthetases and energy regeneration enzymes (PURExpress solution B). A third contained the plasmid. In order to optically distinguish the vesicle populations, two vesicle types were labelled with Cy5-PE and Rh-PE (Cy5 and TRITC filers) and the third was left unlabelled. A network of vesicles (5 ± 2 µm diameter each) was formed with 0.25 M NaCl externally and 0.5 M sucrose internally. The three compartments were fused, and GFP expression followed over time (FITC filter; 1 s exposure). GFP production was detected 20 min after fusion, with the reaction proceeding for ca. 100 min before plateauing (Fig. 9b). These results suggest that the high salt conditions needed to form a VIM do not prohibit biochemical reactions from being performed in the vesicle interior due to the shielding effect of the lipid membrane. The variability in the results (Supplementary Note 13 and Supplementary Fig. 11) is likely due to variability in both vesicle volumes and encapsulation efficiencies of the individual components in the cell-free expression mixture^52,53^.

## Discussion

The flexibility of this platform is derived from the use of optical tweezers, which have previously been used to transport vesicles in space^54,55^, as well as in the generation of droplet networks^56^. Optical tweezers are non-invasive thus eliminating the possibility of cross-contamination, and they can be turned on and off on-demand, and used to manipulate objects in 3-D space with submicrometre resolution.

The structures we form have several interesting properties and potential applications. First, the fact that networks can be modulated and indeed disassembled based on external stimuli (salt and temperature) is a feature which could prove useful for sensing applications and in the construction of responsive systems.

Second, the structures we form are biomimetic and can be used as simplified models in the study of cell biology, with tethers and VIMs being synthetic analogues of neural axons and synapses. VIMs have similar length scales to cellular junctions, and the tethers closely resemble lipid membrane nanotubes, also called tunnelling nanotubes^57^, which bridge distinct living cells for chemical exchange and signalling purposes. As in our experiments, some types of tunnelling nanotubes are not open-ended tethers, instead containing an anchor point where the membranes of the two connected cells meet^58^. In this condition, exchange of membrane or cytosolic contents by simple diffusion across the nanotubes is forbidden while the anchor point can exhibit high mobility. Tether formation also raises the possibility of selective inter-compartment communication across large distances. We note that the structures we form differ from previously reported open nanotube-linked vesicles^19,20,59^ formed using micromanipulators, in that in our case there is no free exchange of material between these compartments; the internal volumes of the vesicles remain distinct. The technological basis of our approach is also conceptually different.

Finally, the demonstration of on-demand vesicle fusion to initiate biochemical reactions shows that our system can serve as a platform with which chemistry and biochemistry are performed in miniaturised picolitre vesicle reactors. The use of lasers to induce fusion means defined vesicles carrying reactive cargoes can be brought together with spatiotemporal precision, and the use of near-infrared light limits damage to biological material. In an analogy to droplet microfluidics, this paves the way for vesicle microfluidics, where vesicles are the basic functional unit on which operations are performed. Vesicles have the added advantage of being in an oil-free environment and providing a delineating membrane as a surface which can be decorated with cellular machinery, e.g., for influx/efflux of materials. They are also more physiologically relevant and can be interfaced with cells and tissues as they exist in a bulk aqueous environment.

At present, there are several limitations associated with our system. As the method relies on individually manipulating compartments, it is only suited for the construction of networks composed of a limited number of vesicles. In addition, although the networks are partially reconfigurable, once connections are made, it is not possible to break them without diluting the salt. Therefore, although it is possible to go from a linear to a square to a pyramidal geometry, the reverse is not possible. Our system also depends on the presence of >0.2 M NaCl to mediate adhesion and relies on sucrose being encapsulated internally for effective trapping. This might not be compatible with some physiological systems, and may change the biophysical properties of the membrane, e.g., its fluidity^60^. To remove the dependence on salt, future systems could employ biotin/streptavidin conjugation, or DNA binding/recognition moieties that are anchored into the vesicle membrane^24^. A further constraint is that for inter-compartment communication, although leakage to the external environment was slowed by the blocker, release was still observed within 10 min and material escaping through the intermembrane space could also not be discounted. With the design of engineered double-membrane spanning proteins^61^ and DNA origami constructs in the future^62,63^, this system could be optimised further. These could also be used to facilitate material transfer through tether-linked vesicles, a functionality that is currently absent.

In conclusion, by engineering a system composed of adherent vesicles that can be manipulated in space using optical tweezers, we were able to connect individual vesicles into networks of user-defined architectures. These networks were reconfigurable which enabled transformations between one geometry to another, and their morphology could be tuned using both physical and chemical variables. Chemically distinct compartments could also be linked across a range of length scales (through five orders of magnitude) using closed lipid tethers derived from both vesicles. By incorporating protein machinery and gold nanoparticles, both inter-vesicle communication and vesicle fusion were achieved, allowing us to mix vesicle material and to trigger cell-free protein expression. The described platform ushers in the possibility of using vesicle networks as models of cellular networks in synthetic biology, for the manufacture of synthetic synapses and gap junctions, as miniature biochemical reaction vessels, and as soft microsystem devices existing in physiological environments. Such systems could also be valuable as models in the study of cell–cell adhesion^64^, particularly in investigating the role of the bilayer itself in this process.

## Methods

### Materials

All lipids were purchased from Avanti Polar Lipids (Alabaster, AL) as powders and used without further purification. Lipids used include POPC (catalogue number 850457); 18:1 Rh-PE(catalogue number 810150); 18:1 NBD-PE (catalogue number 810145); 18:1 Cy5-PE (catalogue number 810335); 16:0 Biotinyl Cap PE (catalogue number 870277). Lipid mixtures were prepared by co-dissolving the required molar ratios of lipids in chloroform. BSA (≥99% assay, catalogue number A4161), α-HL (catalogue number H9395, 10,000 units mg^−1^ protein), calcein (catalogue number C0875), TRIMEB (98% purity; catalogue number H4645) and all buffer reagents were purchased from Sigma-Aldrich (Gillingham, UK). Fluo-4 (pentapotassium salt; 99% purity; catalogue number F14200) was purchased from Thermo Fisher Scientific (Loughborough, UK). A summary of materials and conditions used in the individual experiments are given in Supplementary Table 3.

### Vesicle preparation

Unless otherwise specified vesicles were prepared by electroformation. First, lipid in chloroform (20 µl; 1 mg ml^−1^) was spread evenly on a conductive indium tin oxide coated (ITO) slide, leaving a film which was dried under vacuum for 30 min to remove residual solvent. A 5 mm thick polydimethylsiloxane (PDMS) spacer with a central cut-out was used to separate the slides with the conductive sides facing each other, and the chamber was filled with 1.5 M sucrose solution in DI water. An alternating electric field (1 V, 10 Hz) was applied across the ITO plates using a function generator (Aim-TTi, TG315). After 2 h, the electric field was changed to 1 V, 2 Hz for a further hour, and the resulting vesicles collected.

In the inter-vesicle material transfer experiments, the emulsion transfer method^65^ was used to form vesicles. This was because encapsulating large, charged molecules is not feasible in electroformation. Briefly, 25 µl of 1.5 M sucrose in buffer (500 mM KCl, 25 mM Tris-HCl, pH 8.0) was added to 250 µl mineral oil with dissolved lipid (10 mg ml^−1^). A water-in-oil emulsion was made by vortexing this mixture for 30 s and left standing for 10 min to allow a lipid monolayer to effectively stabilise the emulsion. Then, 250 µl of the emulsion was layered above 250 µl of 1.5 M glucose in buffer solution, forming a water/oil column. This was centrifuged (9000 × *g*, 30 min) resulting in a vesicle pellet. The upper oil phase was removed, and a second centrifugation step was applied (6000 × *g*, 10 min), followed by removal of the supernatant and resuspension in 250 µl fresh glucose solution.

### Network assembly

A coverslip surface was coated with a BSA monolayer to prevent interactions between the glass substrate and the vesicle membrane. The coating was applied by depositing 300 µl of 1% BSA in DI water on a coverslip and leaving it to evaporate in a 60 °C oven, leaving behind a protein film. The film was subsequently rinsed with DI water and dried under a nitrogen stream. Vesicle assembly chambers were prepared by placing a 1 mm thick PDMS sheet with a 10 mm diameter hole on the coverslip. An external solution containing 0.83 M NaCl was added to the vesicle emulsion at a ratio of 9:1, to give a final concentration of 0.75 M. For fusion experiments, the final NaCl concentration used was 0.25 M to osmotically match the 0.5 M sucrose in the vesicle interior. The sample was then mixed by pipette aspiration, the chamber sealed with a second coverslip, and placed on the optical trapping setup. Individual vesicles were trapped by switching the laser on, and were moved in 3-D relative to the sample by moving the microscope stage (*x*,*y*) and changing the focus of the objective (*z*). VIMs were subsequently formed by positioning two or more vesicles into contact. Details of the optical setup are available in Supplementary Note 1

### Vesicle disassembly via salt dilution

In order to dynamically change the NaCl concentration in the vesicle exterior once vesicle networks were generated, the sample well was sealed with an agarose gel (3 wt.% in DI water; 1 mm thick), which allowed diffusion of substances placed above it without disturbing the networks through the generation of large flows. This setup was also used to assemble extended vesicle networks, as it gave time to assemble networks before vesicles would stochastically come into contact with one another to form aggregates.

### Vesicle fusion and vesicle microreactors

To prepare functionalised AuNP vesicles, we first electroformed POPC vesicles in 0.5 M sucrose solution with 2 wt.% 16:0 Biotinyl Cap PE. When fluorescent lipids were present, these were at 1 wt.%. In the calcein dilution and protein expression experiments, vesicles were formed via emulsion phase transfer (see above), with 0.5 M sucrose/glucose density gradient. Next, 150 nm streptavidin-coated AuNPs (Nanopartz, CO, USA; product C11-150-TS-50; 2.5 mg ml^−1^) were added to the vesicles 1:9, with the sample vortexed for 30 min to drive conjugation. VIMs were formed as before, but with 0.25 M NaCl in the external solution. Vesicles were fused by focusing and applying a laser of 150 mW (at trap) or greater on the VIM interface. Vesicles were manipulated in space using a <100 mW laser (at trap) to avoid unintentional fusion (Supplementary Note 13). We also used Aurora-DSG Nanoparticles (AuNP-labelled lipids; 1–2 nm; Avanti), which did not result in fusion when embedded in the membrane at 2 wt.%.

In the protein expression experiments, we used an *E. coli* pJexpress 441 vector (50 ng µl^−1^) with a T7 promoter expressing the fluorescent protein Dasher GFP (ex = 510 nm, em = 521 nm) supplied by ATUM (CA, USA). The PURExpress kit components (New England Biolabs, MA, USA) were assembled according to the manufacturer’s instructions. The kit components and plasmids were made up to 500 mM sucrose by adding appropriate volumes of 2 M sucrose. PURExpress Solutions A and plasmid were prepared in PBS, and Solution B was prepared 10 mM magnesium acetate PBS on the recommendation of the supplier. Vesicles were formed with emulsion phase transfer, with these three solutions being the inner phase. In all fluorescence experiments, total fluorescence of vesicle interiors was divided by the vesicle area, and the background fluorescence subtracted. Fluorescence values were obtained using ImageJ analysis software as grey values.

### Data availability

All relevant data are available from the authors on reasonable request.

## Electronic supplementary material

Supplementary Information

Description of Additional Supplementary Files

Supplementary Movie 1

Supplementary Movie 2

Supplementary Movie 3

Supplementary Movie 4
