# Supplementary material for: Sculpting and fusing biomimetic vesicle networks using optical tweezers
Source: Nat Commun. 2018 May 14;9:1882. doi: 10.1038/s41467-018-04282-w (PMC5951844; doi:10.1038/s41467-018-04282-w)
Supplement: Supplementary file 1 — Supplementary Information [file 41467_2018_4282_MOESM1_ESM.docx]

**Sculpting and fusing biomimetic vesicle networks using optical tweezers**

**Bolognesi *et al.***

**Supplementary Note 1**

**Optical trapping microscope.** An inverted epi-fluorescence dual-carousel microscope (Nikon TE2000-U) combined with an optical trapping system was used for imaging and optical manipulation as described in previous publications. ^1, 2^ Briefly, a linearly polarised beam from an Ytterbium fibre laser source (20 W at 1070 nm; ; IPG Photonics, Europe) was expanded using a pair of IR doublets (f = 200mm, f = 160mm) in order to fill the 20 mm × 20 mm 1280x1024 pixel display of a ferroelectric liquid crystal on silicon (FLCOS) spatial light modulator (SLM) (CRL-Opto SXGA-R-H1). The waist of the beam from the SLM was adjusted by means of a second pair of IR doublets (f=40mm, f=100mm) in order to slightly overfill the back-aperture of 60x 1.2 N.A. water immersion objective mounted on the inverted microscope. A IR dichroic mirror and a IR filter (Chroma, USA) were inserted in the upper carousel of the microscope to direct the trapping beam towards the objective while preventing any beam reflection from reaching the CCD camera (ORCA-ER Hamamatsu). Two Nikon filter cubes (FITC and TRITC), inserted in the lower carousel, together with a mercury-fibre illuminator (Nikon Intensilight CHGFIE) were used for imaging the vesicles in fluorescence mode. The image acquisition process was controlled using a customised Labview (National Instruments Corp) interface. Two half-wave plates, placed one before and one after the SLM, were oriented so that the transmitted beam was no longer modulated by the SLM. Consequently, the latter acted as a simple mirror and the unmodulated (0th order) beam was used to power the single optical trap. The optical manipulation of vesicles typically required a laser power of 80 mW - 470 mW at the back aperture of the objective (23 mW-190 mW at trap).

For cell fusion experiments a conventional single beam optical trapping setup in replacement of the holographic optical trapping system was used. A bespoke 30 mm cage optic and filter cube mount was machined and replaced the upper carousel of the dual-carousel microscope (Nikon TE2000-U). The laser collimator head was mounted in a cage plate and the beam was expanded to fill the back aperture of a 60× 1.4 NA oil-immersion objective with a pair of opposing plano-convex lenses in a Keplerian arrangement along a cage optic rail. The IR dichroic mirror and IR filter (Chroma, USA) were fitted in a filter cube which was mounted in the bespoke mount. The laser power at the back aperture was measured to be 9.9 ± 0.2 % of the nominal laser launch power. The laser focal point was aligned to coincide with the object plane and objects were manipulated by translation of the motorised microscope XY stage.

**Supplementary Note 2**

**Notes on refractive index.** Stable optical trapping of vesicles is a challenging task since these objects typically have very low refractive index contrast with respect to the surrounding medium. One approach to overcome this obstacle is to insert dielectric particles inside the vesicles or within their membranes^3^. The dielectric particles can be hence trapped efficiently by optical tweezers, thereby acting as handles for vesicle manipulation. In this paper, we followed a different method which consists in doping the liquid of the vesicles interior with membrane-impermeable additives (e.g sucrose or glucose) in order to enhance the refractive index contrast of the vesicle. This approach was successfully used in previous studies to control the vesicle shape via multiplexed optical trapping for membrane microrheology analysis^4^ as well as to immobilise nanometer-sized liposomes^5^. It is worth noting that such additives may lead to osmolarity gradients between the external and internal liquid phases which would lead to a net flow of water across the vesicles membrane. This could result in the uncontrolled variation of the vesicle volume, thereby causing unwanted modifications of the vesicle refractive index due changes in the additive concentration within the vesicles. To avoid this, the external and internal liquid phase must have the same osmolar concentration whilst maintaining a refractive index contrast suitable for optical trapping. In our experiments, these conditions were met by using a 0.75 M NaCl aqueous solution and 1.5 M sucrose solution for the external and internal phases respectively. By relying on the literature data^4^ for the refractive index of sucrose and NaCl aqueous solutions at varying salt concentrations and by using the Lorentz-Lorenz formula^6^, the refractive indexes for the external and internal solutions were estimated to be 1.406 and 1.342, respectively. As demonstrated in our experiments, this refractive index contrast is enough to allow for facile and stable trapping and manipulation of vesicles.

**Supplementary Note 3**

**Vesicle adhesion in different conditions.** Larger NaCl concentrations led to larger VIM interfaces (Supplementary Fig. 1). Adhesions lengths between POPC GUVs (1 wt. % Rh-PE) at external NaCl concentrations of 0.2 M – 0.7 M were measured, in increments of 0.1 M. GUVs contained 1.5 M sucrose internally, and varying concentrations of glucose, in addition to the salt, to maintain an osmotic balance.

Increasing vesicle charge led to an increase in the minimum concentration of NaCl needed for adhesion to be observed. Charged DOPG lipids were incorporated into POPC vesicles at different mol % and the minimum concentration of NaCl where adhesion was observed in an osmotically balanced system (1.5 M sucrose internally, varying concentration of NaCl and glucose externally) was measured. This was determined by bringing vesicles into contact using the traps and then dragging them apart. Where dragging one GUV led to the movement of the adjacent GUV the vesicles were deemed to have adhered. NaCl concentration was varied at 0.05 M increments. The more charged lipid was incorporated, the more NaCl was needed to achieve adhesion (Supplementary Table 1). This is a further strand of evidence to suggest that electrostatic screening is responsible for adhesion.

As a further indication that electrostatic effects were the main driving force for vesicle adhesion, we were able to observe VIM being formed with divalent ions (CaCl_2_  and MgCl_2_) at significantly lower concentrations ( 5 mM and 20 mM respectively; these concentrations were 40 and 10 times lower than the concentration of NaCl needed for adhesion). This trend has been reported previously.^7, 8^ These values were found using POPC vesicles (1 wt.% Rh-PE) with 1.5 M sucrose internally and 1.5 glucose with salt present externally.

We ruled out that the presence of fluorescent lipids was responsible for adhesion by removing these from our system. VIMs, visualised using phase contrast microscopy, could still be formed with external NaCl concentrations as low as 0.2 M (1.5 M sucrose internally, 1.1 M glucose externally).

Finally, changing pH didn’t appear to block VIM formation. Vesicles were formed as before, with 1.5 M sucrose internally, and varying concentrations of glucose and NaCl externally to maintain an osmotic balance. Vesicles were formed with (i) TRIS buffer at pH 7 (ii) TRIS buffer at pH 9, (iii) phthalate buffer at pH 9. Irreversible adhesion was seen in all cases at 0.2 M NaCl. This was partly expected as the charge on the membrane is not expected to change at these pHs given the pKa of the lipid. ^9^

**Supplementary Note 4**

**Fluorescence contours.** In the main text, a fluorescence profile of a VIM across two fluorescent vesicles was used as one strand of evidence to support the existence of two adherent bilayers, as opposed to one hemifused bilayer. To exclude any height integration effects (for example from the adhered bilayers, which are flat vs the non-adhered portion of the vesicle which is curved) we performed a control experiment where one POPC vesicle contained 1 wt. % Rh-PE and the other did not. Vesicles (1.5 M sucrose internally), were incubated in 0.75 M NaCl, VIMs were formed, and a fluorescence profile through their midline was taken (Supplementary Fig. 2). The fluorescence intensities of the VIM bilayer and the non-VIM bilayer were the same. We therefore conclude that integration of fluorescence signal over height is not a factor in our experiments, and that the larger fluorescence in the VIM seen in Fig. 4B in the main manuscript is due to the presence of two bilayers and not due to a flat vs. curved membrane.

**Supplementary Note 5**

**Adhesion energy model discussion.** According to the model developed by Chiruvolu et al.^10^, the free energy of two identical adhering vesicles – interacting via non-specific forces – is given by

$$E\left( \theta\right)=4\pi R^{2}\left[ Kf\left( \theta\right)^{2}-\frac{W(1-\cos\theta)(1+f\left( \theta\right)}{2-\cos\theta} \right]$$

(Supplementary Equation 1)

with $\theta$ is the contact angle between the two vesicle membranes, $R$ the radius of spherical vesicle before binding, $K$ the elastic expansion area modulus of the membranes and $W$ the work of adhesion. The function $f\left( \theta\right)$ gives the ratio of the vesicle surface area increase, due to membrane adhesion, over vesicle original area and it can be written as

$$f\left( \theta\right)=\frac{(3-\cos\theta)}{2^{2/3}({1+\cos\theta)}^{1/3}{(2-\cos\theta)}^{2/3}}-1$$

Eq. (Supplementary Equation 1) holds in the strong-adhesion limit for which the bending energy is negligible compared to the adhesion and elastic stretching energies. In our experimental conditions, the dimensionless adhesion potential^11^ $WR^{2}/\kappa$ (with $\kappa$ the bending rigidity) is on the order of ${10}^{-6}$, hence the strong-adhesion assumption is valid.

(Supplementary Equation 2)

The equilibrium contact angle $\theta_{0}$ is given by the following condition

$$\text{For} \theta=\theta_{0} : \left( \frac{\partial E}{\partial\theta} \right)_{V}=0$$

(Supplementary Equation 3)

where the subscript $V$ denotes that the derivative is calculated at constant vesicle volume $V$ and, hence, constant radius $R$. The equilibrium interaction energy $E_{0}=E(\theta_{0})$ is well approximated by the following relation^10^

$$E_{0}\simeq\frac{\pi^{2}R^{2}W^{4/3}}{{4^{1/3}K}^{1/3}}$$

(Supplementary Equation 4)

By solving Eq. (Supplementary Equation 3) with respect to the adhesion energy $W$, we obtain

$$W=2K\left( 1-\cos\theta_{0} \right) f(\theta_{0})$$

(Supplementary Equation 5)

By inserting the equilibrium membrane tension $\sigma_{0}=Kf(\theta_{0})$ in Eq. (Supplementary Equation 5), the well-known Young equation is obtained

$$W=2\sigma_{0}\left( 1-\cos\left( \theta_{0} \right) \right)$$

(Supplementary Equation 6)

For small values of $W/K$, the equilibrium angle $\theta_{0}$ can be approximated through a perturbation expansion of Eq. (Supplementary Equation 5) in terms of $W/K$, which leads to Equation (1) in the manuscript

$$\cos\theta_{0}\simeq1-\left( \frac{2W}{K} \right)^{\frac{1}{3}}$$

(Supplementary Equation 7)

Eq. (Supplementary Equation 7) provides a good approximation for the solution of Eq. (Supplementary Equation 5) under the examined experimental conditions. For W = 1 mJ/m and K = 200 mJ/m, Eq. (Supplementary Equation 7) gives $\theta_{0}=38.2^{\circ}$ whereas the actual solution, calculated by numerically solving Eq. (Supplementary Equation 5), is $\theta_{0}=39.3^{\circ}$.

Eq. (Supplementary Equation 1) was obtained by making the following two assumptions: i) the vesicles are initially unstressed and ii) the vesicles volume is constant. In principle, condition i) is not met since before binding the vesicle do have a residual interfacial stress due to a non-vanishing surface tension. Nevertheless, the elastic membrane deformations caused by the vesicle binding process result in membrane elastic tension $\sigma$ at the equilibrium of the order of few mN/m. As a consequence, the membrane tension of unbound vesicles is negligible compared to those achieved after adhesion and, hence, condition i) is satisfied. To verify conditions ii), the vesicle volume variation induced by the membrane elastic deformation is estimated as follows. The pressure increase of the liquid within the vesicle caused by the vesicle deformation is given by

$$\Delta p= \frac{2 \sigma}{R}$$

(Supplementary Equation 8)

For $\sigma$=1mN/m and $R$=10 µm, it follows $\Delta p$=200 Pa. According to van’t Hoff formula, an osmotic concentration increase $\Delta c=\frac{\Delta p}{R T}=$80mM is generated at room temperature within the vesicle to balance the Laplace pressure $\Delta p$. Since the sucrose cannot permeate the membrane, water leaks out the vesicles until the sucrose concentration is increased by $\Delta c$ at equilibrium. Since the number of moles of sucrose within the vesicles is conserved (i.e. $V_{i}c_{i}=V_{f}c_{f})$, the corresponding relative volume decrease is calculated as

$$\frac{V_{i}-V_{f}}{V_{i}}=\frac{c_{f}-c_{i}}{c_{f}}=\frac{\Delta c}{c_{i}+\Delta c}$$

(Supplementary Equation 9)

the subscript i and f referring to the status before and after binding, respectively. Since the initial sucrose concentration is equal to $c_{i}=$1.5 M, the vesicle relative volume decrease is ca. $5\%$. We conclude that under the examined experimental conditions, the vesicle volume variations are relatively small and, hence, condition ii) holds.

Finally, it worth noting that this model assumes identical vesicle size and properties (e.g. tension, expansion modulus, etc.) and, under such assumptions, the intermembrane adhesion patch is flat. As a consequence, for the purpose of measuring the adhesion energy from contact angle analysis, only similarly sized vesicle pairs with a flat contact area were considered.

**Supplementary Note 6**

**Contour extraction algorithm.** The contact angle is defined as half the angle between the tangent lines of both vesicles from the ternary intersection as shown in Supplementary Fig. 3. Note that the tangent line from the interface can be curved in cases where the vesicle volumes are mismatched.

The angle $\theta$ can be found from the law of cosines, by the equation

$$2\theta={180}^{^{\circ}}-\mathrm{acos} \left( \frac{\left( r_{1} \right)^{2}+\left( r_{2} \right)^{2}-h^{2}}{2r_{1}r_{2}} \right)$$

(Supplementary Equation 10)

The vesicle radii and centres of the grey threshold binary images were found using a Hough transform from the MATLAB image processing toolbox. The image was converted to greyscale and filtered with Contrast-Limited Adaptive Histogram Equalization. The filtered image was rotated to orientate the vesicle pair horizontally and the vesicle interface is found from the intensity peaks. This interface could be linear (Supplementary Fig. 3D) or curved towards the smaller vesicle (Supplementary Fig. 3E) when the vesicle volumes were mismatched. The overlay fit circles are shown in the original vesicle images in the figures for clarity.

**Supplementary Note 7**

**Intervesicle adhesion and nanotube formation forces.** The interactions between the membranes of two adhering vesicles is equivalent to a force $\boldsymbol{F}_{\boldsymbol{x}_{\boldsymbol{C}}}$ applied to the vesicle centres and perpendicular to the adhesion area. The intensity of $\boldsymbol{F}_{\boldsymbol{x}_{\boldsymbol{C}}}$ is given by

$$F_{x_{C}}=\left( \frac{\partial E_{ves}}{\partial x_{c}} \right)_{V}$$

(Supplementary Equation 11)

where $E_{ves}=\frac{1}{2}E$ is the interaction energy of one single vesicle and $x_{c}=r\cos\left( \theta\right)$ is the distance between the vesicle centres and the intermembrane adhesion area (see inset in Supplementary Fig. 4). Since the total interaction energy $E$ is given by the sum of the elastic energy of each vesicle $E_{el}$ and the intermembrane adhesion energy $E_{ad}$, it follows that

$$E_{ves}=E_{el}+\frac{1}{2}E_{ad}$$

(Supplementary Equation 12)

By combining Eq. (Supplementary Equation 11) and (Supplementary Equation 12), it results

$$F_{x_{C}}=\left( \frac{\partial E_{el}}{\partial x_{c}} \right)_{V}+\frac{1}{2}\left( \frac{\partial E_{ad}}{\partial x_{c}} \right)_{V}=F_{el}+F_{ad}$$

(Supplementary Equation 13)

where $F_{el}=\left( \frac{\partial E_{el}}{\partial x_{c}} \right)_{V}$is the elastic force induced by the elastic response of the deformed vesicles and $F_{ad}={\frac{1}{2}\left( \frac{\partial E_{ad}}{\partial x_{c}} \right)}_{V}$is the adhesion force due to the vesicle membrane interaction. At the equilibrium, $F_{x_{C}}=0$, so the elastic and adhesion forces are perfectly balanced. For an optically-trapped vesicle, the optical force can be described with an equivalent force $\boldsymbol{F}_{\boldsymbol{trap}}$ applied to the vesicle centre, whose component in the direction perpendicular to the adhesion area is given at the equilibrium by

$$F_{trap}+F_{x_{C}}=0$$

(Supplementary Equation 14)

and, hence,

$$F_{trap}=-\left( \frac{\partial E_{ves}}{\partial x_{c}} \right)_{V}=-F_{el}-F_{ad}$$

(Supplementary Equation 15)

As a numerical example, the adhesion force $F_{ad}$, the elastic force $F_{el}$ and the trapping force $F_{trap}$ are calculated according to Eq. (Supplementary Equation 13) and (Supplementary Equation 15) for a pair of adhering vesicles under similar conditions to those observed experimentally for the generation of vesicle-nanotube networks (i.e. $R=4$µm, $W=0.1$mN/m^2^, $K =213$ mJ/m^2^). As shown in Supplementary Fig. 4, the trapping force must rapidly increase as the contact angle departs from the initial equilibrium value$\theta_{0}\simeq25.8^{\circ}$. At decreasing contact angles, the vesicles shape tends towards the original spherical shape of unbounded unstressed vesicles and, as a result the elastic force decreases as well. The adhesion force is instead approximately constant for any contact angle. At zero contact angle (i.e. for spherical undeformed vesicles), the adhesion force can be calculated by using the Derjaguin approximation, which gives $F_{ad}(\theta=0)=\pi R_{0}W$.

Complete vesicle separation (i.e. $\theta=0$) could be achieved when the trapping force equals the adhesion force on the order of 1 nN. However, the latter value is well beyond the range of optical force amplitudes for continuous laser optical tweezers. Conversely, as the intervesicle distance increases, the trapping forces increases until it reaches the critical value $F_{cr}$ required for the formation of a nanotube. Koster et al. experimentally validated a simple analytical expression for $F_{cr}$ but this was determined under the assumption of constant membrane tension during nanotube formation.^12^

However, in our experiments the membrane tension varies from ca. 1 mN/m in the absence of trapping force (i.e. ${\theta=\theta}_{0}$) to ca. 1 µN/m when the nanothread is formed, so more advanced models would be required to quantify the actual value of $F_{cr}$. Nevertheless, in our optical trapping set-up the amplitude of the photonic force exerted by the laser on the GUVs can range from 0 to ca. 200 pN at full laser power (see grey area in Supplementary Fig. 4). According to our experimental observation, such force intensity range is enough to generate a nanothread under the examined conditions. Critically, the adhesion force remains much higher than the force required to pull and sustain a nanothread even for small adhesion area. This ensures a stable connection at the junction between two adhering nanothreads.

It is worth noting that the adopted model assumes a spherical shape for the non-adhered portion of the vesicle membranes. However, this condition is met only in the absence of external forces. Experimentally, we observed that the non-adhered membrane regions deformed under the effect of photonic forces and their shape were no longer spherical. Even though this behaviour affects the accuracy of our force estimates, the overall physical description of the nanotube formation process remains valid. To conclude, it is noted that the adopted model assumes the adhesion energy to be independent of the membrane tension. However, it is known that energy terms that contributes to the overall adhesion energy $W$, such as hydrophobic attraction and undulation interactions, depends on the tension state of the membrane. The broad range of tension values experienced by the membrane during nanotube formation suggests that the variation in adhesion energy with tension might not be negligible and the adhesion energy between two nanotubes might quantitatively differ from the one calculated between two adhering vesicles.

**Supplementary Note 8**

**Laser-induced heating.** Previous studies have shown how the tightly focused light of an optical trap may result in local heating of both the trapped object and surrounding fluid^13^. To quantify the laser-induced thermal effect in our optical set-up, the total laser power delivered to the liquid sample was first determined, followed by the calculation of the corresponding sample temperature rise. It is worth noting that the trap power is only a fraction of the beam power delivered to the rear aperture of the objective, due to significant losses occurring within the lens. The objective transmittance $T$ was quantified by using a two-objective measurement method, similar to the one described by Misawa et al^14^. In Misawa’s method, two identical objectives are used to focus and recollimate the transmitted laser beam, so that the total transmission for the objectives can be easily determined. Due to the unavailability of second lens identical to the trapping objective, we performed the transmittance measurements by coupling the trapping lens with a 10x (0.3 NA) objective, whose transmittance was previously determined from a direct lens-to-photodetector measurement. The trap power $P_{trap}$ was hence obtained as $T P_{BA}$, where $T$ is the transmittance of the trapping objective and $P_{BA}$ is the beam power measured at the objective back aperture. The measured values of $P_{BP}$ and the resulting values of $P_{trap}$ are reported in Supplementary Table 2. In this range of power levels, the measured objective transmittance $T$ was ca. 40%.

To determine the temperature rise induced by the optical trap at a given power $P_{trap}$, a finite element model was developed in Comsol Multiphysics to solve the heat equation in the stationary regime. The intensity profile of the Gaussian laser beam was modelled according to the paraxial approximation and only the light absorption from liquid water was considered – hence neglecting the absorption at the solid walls of the fluidic cell. The maximum temperature increase ${\Delta T}_{MAX}$ - reached at the trap centre – was numerically calculated for an optical trap located at 10 µm from the bottom wall of a 200 µm deep cell filled with water. ${\Delta T}_{MAX}$ increases up to 7$℃$ for a power trap of 470 mW (see Supplementary Table 2). For optical traps, the heating effect is highly localised and the temperature rapidly decreases at increasing distance from the trap. In Supplementary Table 2, the temperature rise${\Delta T}_{10\mu m}$ calculated at 10 µm distance from the trap centre on the focal plane are reported.

The increased VIM length upon application of the laser is likely due to the temperature increase. This is confirmed by experiments conducted using a temperature controlled heating stage. POPC VIMs (1.5 M sucrose internally, 0.75 M NaCl externally) were generated, and the bulk sample was heated from 21°C – 26°C and cooled back down. A corresponding increase and decrease in VIM length was observed (Supplementary Fig. 5A).

Furthermore, when a laser (0.95 W) was placed away from the vesicle (no longer trapping it), similar changes in VIM morphology was observed (Supplementary Fig. 5B). This was less pronounced as when the laser was trapping the vesicle, due to the heat dissipation effect described above. Since the vesicle pair does not migrate towards the laser focus, the optical forces exerted by the trapping laser can be neglected under this condition. However, although the vesicles are not optically trapped, they are still affected by the temperature gradient created by the tightly focused laser, further suggesting that temperature changes are responsible for this effect.

**Supplementary Note 9**

**Small Angle X-Ray Diffraction.** To determine the intermembrane spacing adjacent bilayers of the VIM, small angle X-ray scattering (SAXS) experiments were performed on bulk POPC at 80 % hydration (fully hydrated) with 0.75 M NaCl in DI water at 25 °C. Following the addition of water, the sample was freeze-thawed ten times with vigorous vortexing between each cycle. It was then placed in a capillary and sealed. SAXS patterns (Supplementary Fig. 6) were collected using a custom built X-ray beamline based on a Bede microsource X-ray generator giving Cu Kα radiation with a wavelength of 1.5406 Å and a Photonic Science Gemstar image intensified CCD based detector. The sample temperature was controlled via a peltier based sample heating device.

As multi-lamellar stacks and adhered GUVs are different systems, the values obtained will not be identical, although they are likely comparable.^15, 16^ Both systems are under full hydration, so the hydration forces will be similar,^16^ and effects for example from having adjacent bilayers on both sides in lamellar stacks are likely to be second-order ones. In terms of undulation forces, undulation in the adhered membranes are dampened due to increased lateral tension. There may also be hydrophobic attractions due to stretching of the membrane, exposing the hydrophobic cores.^15^ Finally, the presence of α-HL is unlikely to affect the intermembrane distance as the bulky mushroom cap of the protein is facing towards the vesicle interior and therefore not interfering with the VIM.

**Supplementary Note 10**

**Closed tethers.** To gain insight into the nature of the tethers linking two vesicles, we performed experiments on tether-linked POPC vesicles to determine if there is free diffusion of lipid material and encapsulated cargo between vesicles (Supplementary Fig. 7), which would indicate an open tether as seen in other systems^17, 18, 19^. In the first experiment tethers were generated as before, but with one vesicle containing calcein (1 mM). The tether was held for 20 minutes by continual application of a laser, and the fluorescence level of both vesicles monitored. No significant change was seen in either vesicle over this time (paired T test; n =5; p < 0.001) demonstrating the lack of free diffusion of encapsulated material. In the second experiment a tether was formed between two vesicle where only one contained 1% fluorescent Rh-PE. Diffusion of fluorescent lipid was not observed over 20 minutes. Indeed, a clearly defined truncation in fluorescence was observed at the mid-tether anchor point where the two sub-tethers met, indicating a lack of continuous membrane. Both these results suggest the presence of two distinct sub-tethers that are closed off from one another, instead meeting an anchor point where they adhere. This is reinforced by the observation that upon removal of the trap the tether retracts, and an adhesive patch between two vesicles reforms. In an open tether system with one continuous membrane, full retraction of the tether would lead to merging of the two encapsulated volumes and the formation of a single vesicle.

**Supplementary Note 11**

**Vesicle communication experiments.** Two types of vesicles were generated using the emulsion phase transfer method.^20, 21^ All aqueous solutions were prepared in buffer (500mM KCl, 25mM Tris-HCl, pH 8.0). First the ‘inner’ vesicle solution was prepared in an Eppendorf. For the first vesicle type, this consisted of CaCl_2_ (200 mM), sucrose (1.5 M), and α-HL (50 ng µl^-1^). For the second vesicle type, this consisted of Fluo-4 (0.54 mM), EDTA (1 mM), and sucrose (1.5 M). The external solution was identical, but with glucose replacing sucrose. 25 µl of the inner solution was added to 250 µl mineral oil with dissolved lipid (10 mg ml^-1^). A water-in-oil emulsion was made by vortexing this mixture for 30 s and left standing for ten minutes to allow a lipid monolayer to effectively stabilise the emulsion. 250 µL of the emulsion was then layered above 250 µl of external solution, forming a water/oil column. This was then centrifuged (9000 g, 30 min) resulting in a vesicle pellet. The upper oil phase was then removed and the pellet resuspended. Immediately prior to the experiment, external Ca^2+^/Fluo-4 was removed from the system by forming a pellet *via* centrifugation (6000 g, 10 minutes), removal of supernatant, and re-suspending in fresh buffer containing TRIMEB (10 mM). This prevented efflux of Ca^2+^ out the system. This process was repeated three times. Both GUV types were then brought together to form a VIM as described in the text. Vesicles were always formed on the day, and purified no more than two hours before the start of the experiments. As α-HL inserts into the membrane in a defined orientation, with the ‘mushroom cap’ forming on the same side as where the protein monomers are originally present.^22^ The blocker binding site is on the opposite side to cap.^23^ For this reason the system was designed so that α-HL was present in the internal vesicle volume and blocker in the vesicle exterior.

Once vesicles were brought together (t = 0 min) an increase in fluorescence was observed as Ca^2+^ translocated to the dye-containing compartment (FITC filter; 100 ms exposure; Supplementary Fig. 8A). This typically occurred after a lag time of up to 4 minutes, with the lag time defined as the point at which a 5% increase from the initial fluorescence is seen, with the maximum fluorescence being defined as 100%. This lag time has been observed in other systems^24, 25^ and is due to the presence of EDTA within the vesicles, which was originally added to the dye-containing compartment to minimise background fluorescence. As Ca^2+^ flowed into the vesicle, it was first chelated by the EDTA instead of binding to the dye. Only after enough time could this chelation be overcome to yield a fluorescence increase. The data from 5 individual runs are shown below, with each point being the mean grey value of the dye-containing vesicles.

In order to investigate whether Ca^2+^ was moving between the vesicles via the bulk solution, and not through the VIM, we performed a control where vesicles we brought into contact, but no VIM was formed due to the absence of NaCl. One vesicle contained CaCl_2_, (200 mM), sucrose (1.5 M), and α-HL (50 ng µl^-1^) and the other Fluo-4 (0.54 mM), EDTA (1 mM), and sucrose (1.5 M). Glucose (1.5 M) and TRIMEB (10 mM) were present in the exterior. Vesicles were manipulated into contact using the trap as before, but no adhesion was observed. Fluorescence images on the FITC channel were acquired every 30 s with no fluorescence increase observed even after 120 minutes. This indicates that diffusion of Ca^2+^ into the bulk and then back into the adjacent vesicle was not responsible for the fluorescence increase in the vesicle communication experiments.

The success of this experiment was reliant on effective removal of Ca^2+^ which may have been present due to vesicle rupture during the phase transfer process. We confirm that three centrifugation/purification rounds are enough to achieve this by adding Fluo-4 (10 µl; final concentration 0.54 mM) to the POPC vesicle solution (90 µl) 5 hours after vesicle generation and purification. Fluorescence of the bulk solution (FITC channel, 500 ms exposure) was no different to the control scenario (Supplementary Fig. 8B), where fresh buffer instead of vesicle solution was added, demonstrating effective removal of unencapsulated Ca^2+^.

**Supplementary Note 12**

**Effect of TRIMEB blocker on vesicle leakage.** The effectiveness of cyclodextrin blocker (heptakis(2,3,6-tri-O-methyl)-β-cyclodextrin; TRIMEB) blocking Ca^2+^ diffusion through α-HL was confirmed by conducting a leakage assay on POPC vesicles generated by emulsion phase transfer in buffer (500mM KCl, 25mM Tris-HCl, pH 8.0). The vesicle interior contained sucrose (1.5 M), Fluo-4 (0.54 mM) and EDTA (1 mM) which chelated any residual Ca^2+^ that may have been present in the vesicle interior to reduce the noise. The vesicle exterior contained Ca^2+^ (200 mM), glucose (1.5 M) and α-HL (50 ng µl^-1^ mM) which assembles into heptameric pores on the membrane. Fluorescence of individual vesicles were monitored over time (Supplementary Fig. 9). In experiments where TRIMEB (10 mM) was also present in the vesicle interior, the rate of fluorescence increase was significantly reduced relative to when TRIMEB was absent, with fluorescence reaching a maximum approximately five times slower. This confirms successful, if not complete, blocking of the membrane pores. These experiments demonstrate that vesicles leak within 10 minutes, after which point the Fluo-4 becomes saturated, and further influx of Ca^2+^ cannot be detected. However as Ca^2+^ concentration is c. 370 times higher than the dye concentration in the vesicle communication experiments, there is enough Ca^2+^ left in the compartments by time a VIM is formed for inter-compartment communication to occur. This is confirmed by the results in Supplementary Fig. 8.

**Supplementary Note 13**

**Vesicle fusion and cell-mimetic microreactor experiments.** Network assembly followed by laser-mediated VIM fusion was possible due to the different laser powers needed to perform both operations (Supplementary Fig. 10A). This allowed, for example, manipulation of vesicles in the vertical direction to assemble a pyramid (tetrahedron) followed by fusion of the four compartments to yield a single vesicle. Labelling the vesicles with AuNPs didn’t measurably affect the size of the adhesion patch (Supplementary Fig. 10B), suggesting that the energetics of adhesion is not significantly affected by the presence of AuNPs.

Independent curves for GFP expression as result of fusion of vesicles containing PURExpress solution A, PURExpress solution B, and pJexpress 441 vector with a T7 promoter expressing the fluorescent protein Dasher GFP is shown in Supplementary Fig. 11.


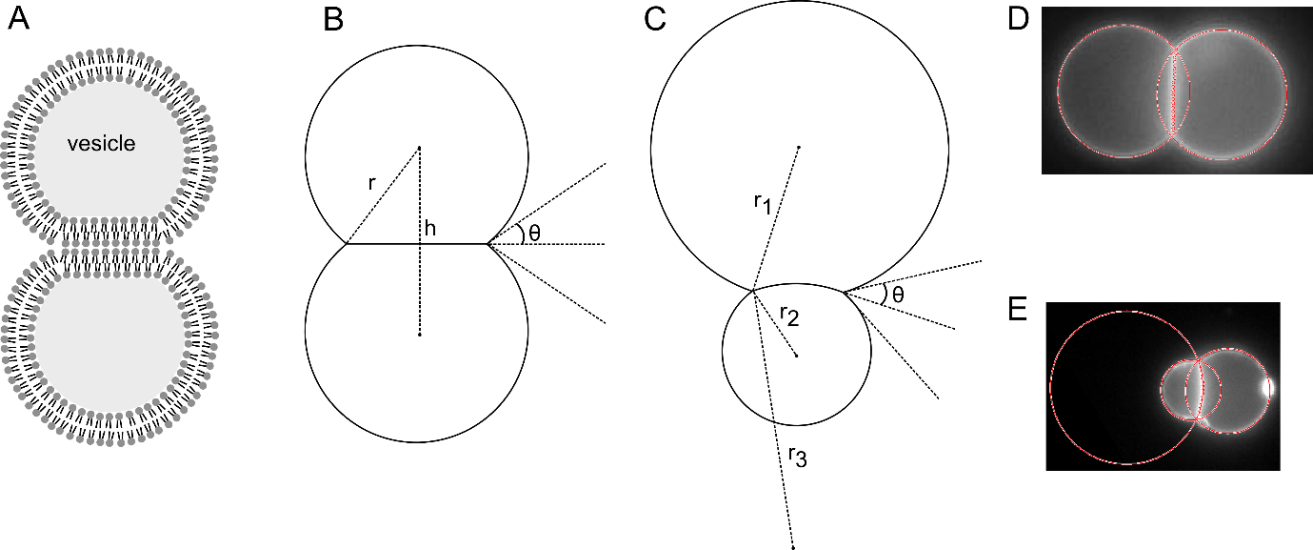


**Supplementary Fig. 3. Geometry of an adhered vesicle pair**. (A) Schematic of two adhered vesicles (B) Geometric parameters associated with two adhered vesicle pairs of identical volumes and (C) of non-identical volumes. (D) Fluorescence image detected VIM contours of two identical volume vesicles and (E) two non-identical volume vesicles.


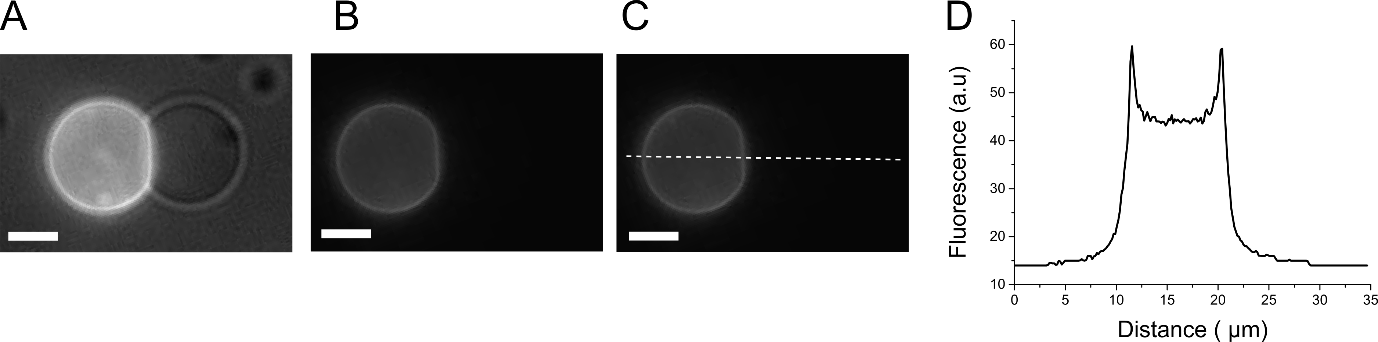


**Supplementary Fig. 2.** (A) Image of adhered vesicle pair where one is fluorescent and the other is not. Fluorescence and brightfield images are superimposed. (B) Fluorescent image of the vesicle pair with (C) a dotted line representing the intensity profile that was taken. (D) Fluorescence profile through the vesicle. The adhered and non-adhered portions of the vesicle membrane show similar fluorescence (c. 60 a.u).


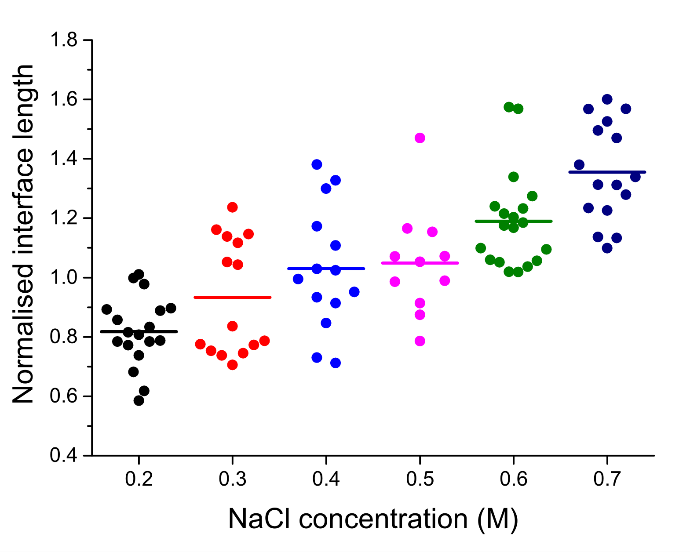


**Supplementary Fig. 1.** Graph showing vesicle interface length between two adhering POPC GUVs at different NaCl concentrations. Each circle represents data from a single vesicle pair, the solid lines represent the mean value.


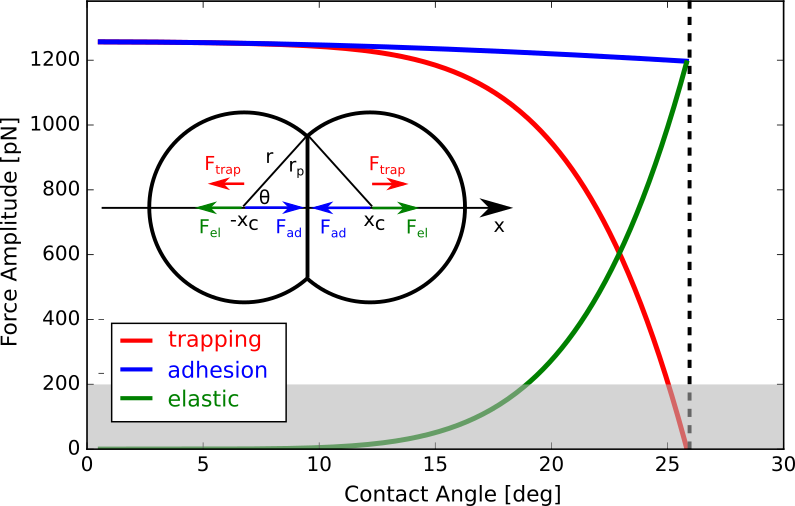


**Supplementary Fig. 4.** Trapping, adhesion and elastic forces at varying contact angles $\theta$ for two identical adhering vesicles with $R=4$µm, $W=0.1$mN/m^2^ and $K =213$ mJ/m^2^. The shaded grey region corresponds to the range of optical forces. The vertical dashed line corresponds to the initial equilibrium contact angle $\theta=\theta_{0}$ achieved in the absence of external forces. A schematic of the VIM morphology together with the forces acting on the two vesicles are shown in the inset.


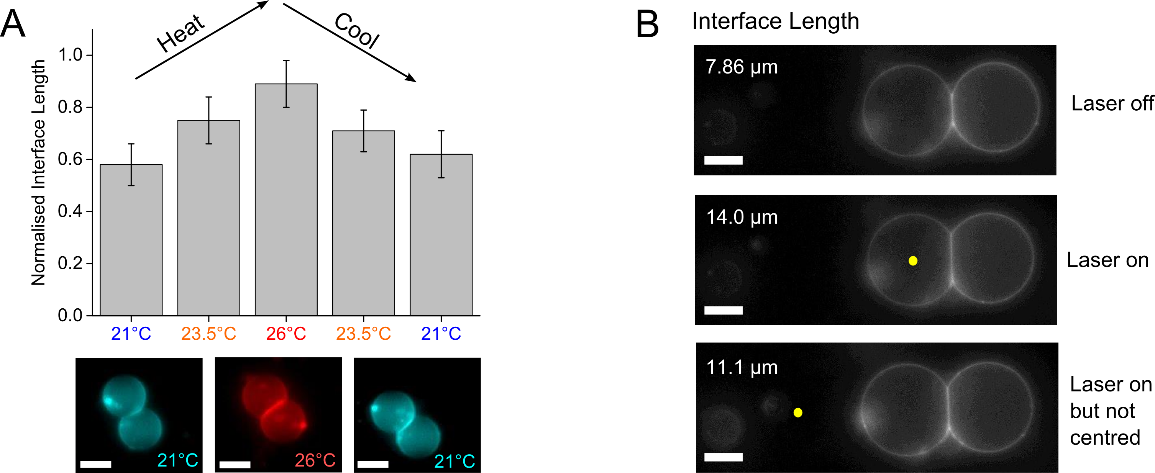


**Supplementary Fig. 5. Temperature effects on VIM length. (A)** Graph showing the change in VIM length as adhered vesicles were heated and cooled using a heating stage. A rise in temperature resulted in a longer interface. Error bars = s.d; n=10. Fluorescence microscopy images of representative adhered vesicles at different temperatures are shown below. **(B)** Fluorescence microscopy images of adhered vesicles, with the laser positioned at different locations (yellow dot). When the laser was placed away from the vesicle, the VIM length was lower that when it was centred, due to heat dissipation. Scale bar = 10 µm for all images.


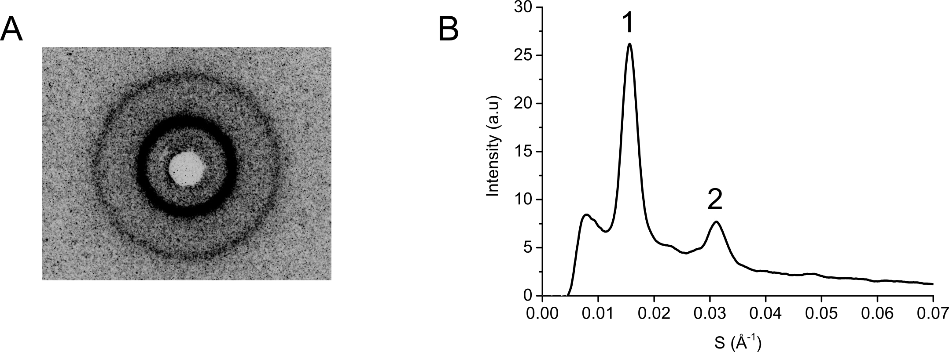


**Supplementary Fig. 6. SAXS of POPC in 0.75 M NaCl**. (A) Contrast-inverted image of scattering pattern showing two clear diffraction rings. (B) Radial integration of the image, displaying two peaks from which a d-spacing was extracted.


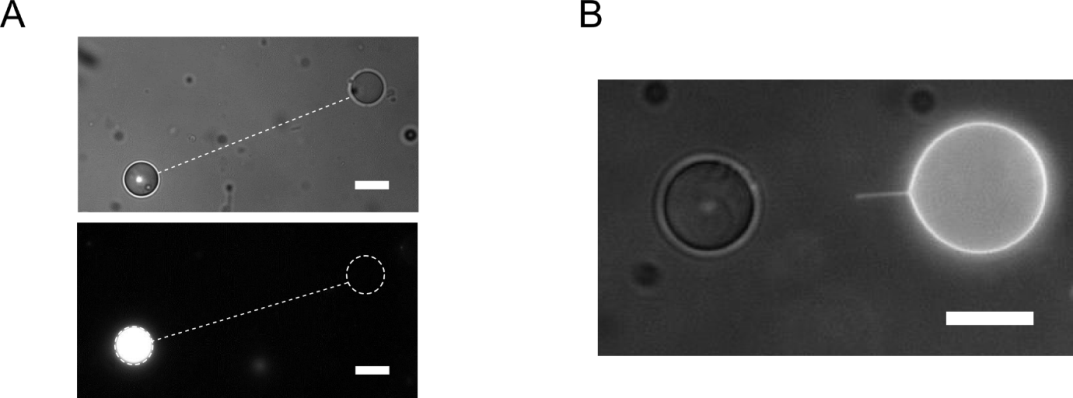


**Supplementary Fig. 7. Fluorescence microscopy images demonstrating closed tethers. (A)** Brightfield and fluorescence images of two vesicles connected by a tether (dotted line) 20 minutes after tether generation. One vesicle contained calcein, the other did not. The lack of fluorescence in the empty vesicle shows that material did not diffuse through the tether **(B)** Brightfield/fluorescence composite image of two tether-lined GUVs, one containing Rh-PE fluorescent lipid (right), the other not (left), 20 minutes after tether generation. The absence of fluorescence in the left vesicle, and the sudden truncation of fluorescence at the anchor point in the middle of the tether, indicates a non-continuous structure, where free diffusion of lipid does not occur. Both these result point to the existence of a closed as opposed to an open tether. Scale bars = 10 µm.


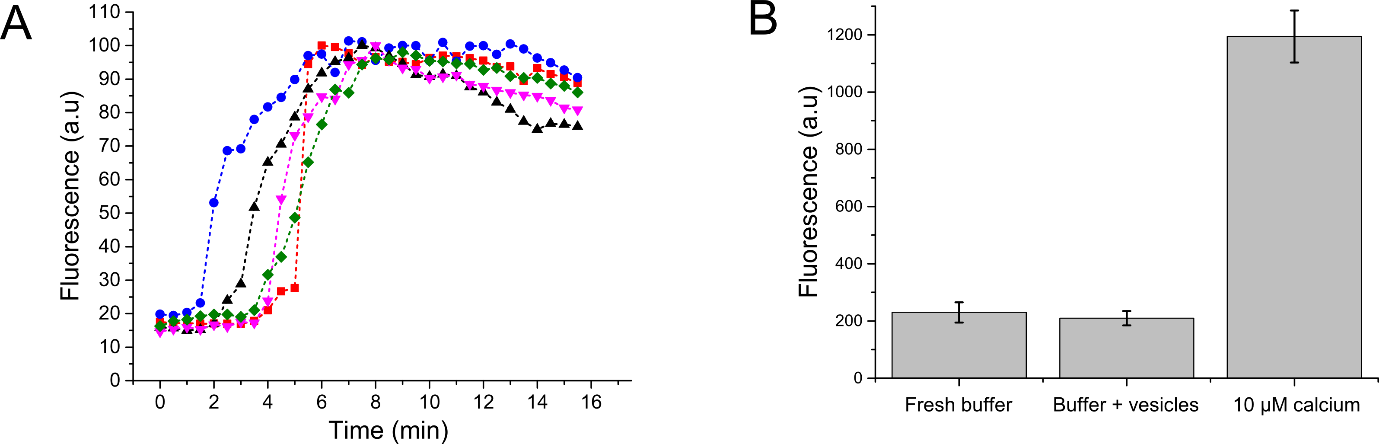


**Supplementary Fig. 8.** (A) Vesicle communication through double bilayer. Graph showing fluorescence levels of five individual vesicles pairs over time, with t = 0 defined as the moment two vesicles adhere to form a VIM. (B) Mean fluorescence of different solutions when Fluo-4 added after 5 hours (n=10; error bars = 1 s.d).


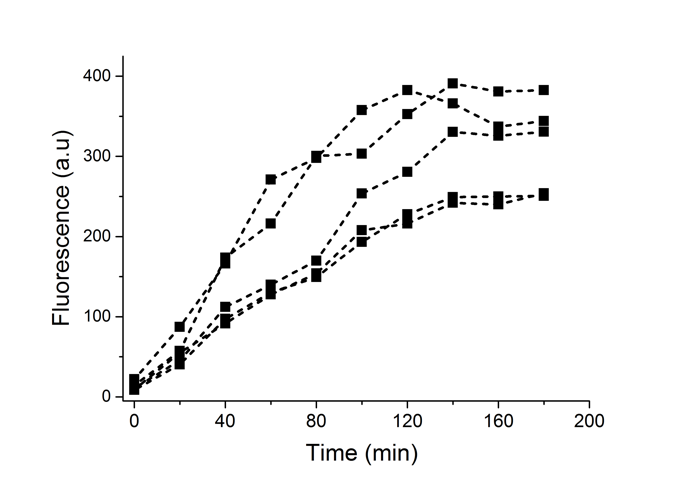


**Supplementary Fig. 11.**  Graph showing fluorescence of fused vesicle over time. An increase in fluorescence was seen after 20 minutes was seen in each case as GFP was synthesised in situ through in vitro transcription and translation.


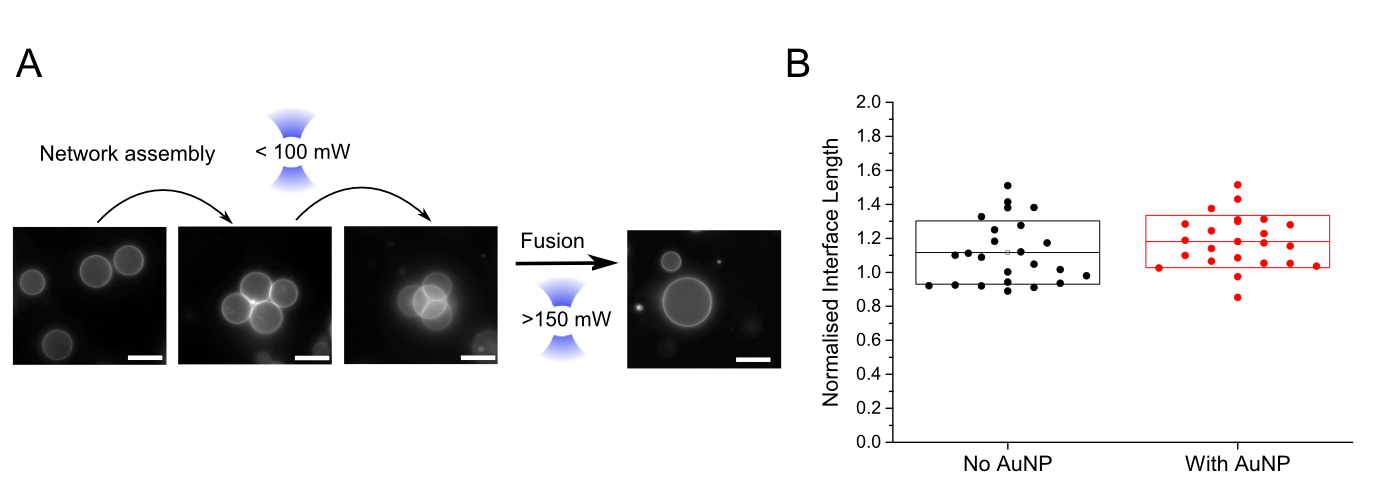


**Supplementary Fig. 10. Vesicle fusion. (A)** Vesicle could be manipulated and assembled in 3D networks and subsequently fused. Inadvertent fusion during manipulation was avoided by using different laser powers for both operations. Laser powers represent power at trap. Scale bars = 20 µm **(B)** Comparison of interface lengths of adhered POPC GUVs and adhered AuNP functionalised POPC GUVs. Vesicles had 1.5 M sucrose internally and 0.75 M NaCl externally. Box represents mean and SD values.


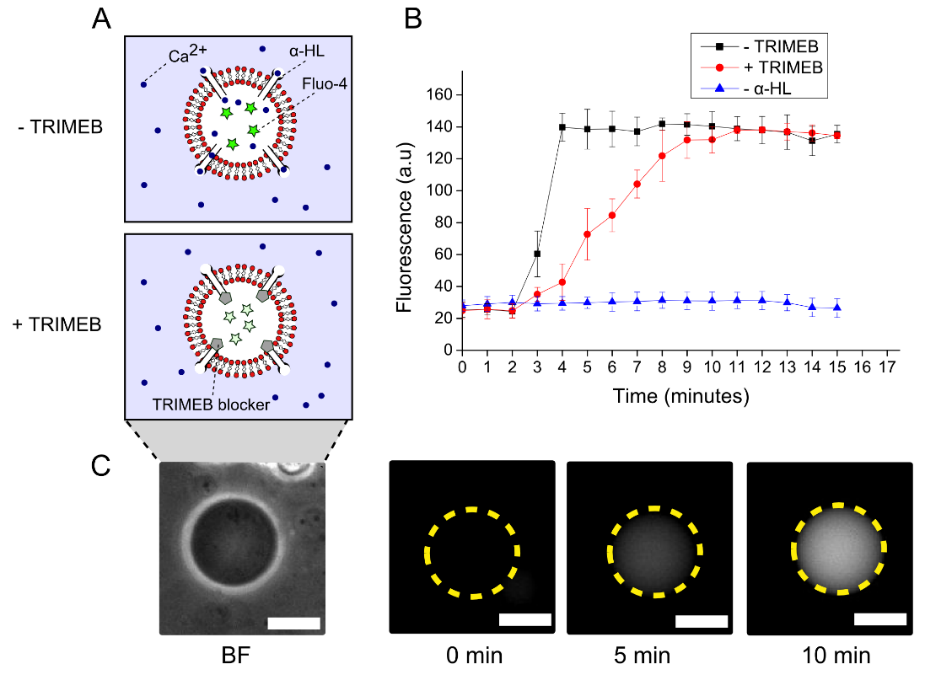


**Supplementary Fig. 9. Effect of TRIMEB blocker on Ca^2+^ leakage into a single vesicle. (A)** Schematic of experiment, in the absence and presence of blocker **(B)** Graph of vesicle fluorescence over time (n= 5; error bar = 1 S.D.), showing that the TRIMEB blocker is effective at slowing the rate of Ca^2+^ diffusion through α-HL. **(C)** Brightfield and fluorescence timecourse image of a typical vesicle, showing an increase fluorescence over time. Dotted yellow circle represents vesicle boundary.

**Supplementary Table 1.** Comparison of minimum NaCl concentration needed to achieve adhesion with POPC GUVs (1 wt. % Rh-PE) with varying amounts of charged lipid incorporated. ^*^

| **Mol % DOPG (charged)** | **Minimum [NaCl] needed for adhesion** |
| --- | --- |
| 0 | 0.2 M |
| 4 | 0.25 M |
| 8 | 0.3 M |
| 12 | 0.4 M |
| 16 | 0.55 M |
| 20 | 0.7 M |

^*^ The more charged lipid present the more salt is needed to dampen electrostatic repulsion for adhesion to occur.

**Supplementary Table 2.** Measured power levels at the objective back aperture and trap centre, and calculated temperature rise at the trap centre and at 10 µm distance.

| **Power Levels [mW]** | | **Temperatures [**$\mathbf{℃}$**]** | |
| --- | --- | --- | --- |
| $P_{BP}$ | $P_{TRAP}$ | ${\Delta T}_{MAX}$ | ${\Delta T}_{10\mu m}$ |
| 80 | 22.9 | 0.4 | 0.1 |
| 350 | 127 | 1.9 | 0.7 |
| 660 | 278 | 4.2 | 1.5 |
| 950 | 367 | 5.6 | 1.9 |
| 1200 | 471 | 7.2 | 2.5 |

**Supplementary Table 3.** Summary of conditions used

| Experiment | Vesicle method of formation and composition | Composition of vesicle interior and exterior |
| --- | --- | --- |
| Vesicle network assembly | Electroformation  POPC 1 wt.% Rh-PE | DI water  1.5 M sucrose internally  0.75 M NaCl externally |
| Dynamic network modulation using salt | Electroformation  POPC 1 wt.% Rh-PE | DI water  1.5 M sucrose internally  0.2 M – 0.75 M salt externally |
| Vesicle adhesion length as a function of NaCl concentration | Electroformation  POPC 1 wt.% Rh-PE | DI water  1.5 M sucrose externally  Varying NaCl concentrations (0.2-0.7 M) osmotically balanced with glucose (0.1 – 1.1 M) |
| Minimum NaCl concentration as a function of charged lipid content | Electroformation.  POPC 1 wt.% Rh-PE, varying mol % of DOPG | DI water  1.5 M sucrose internally  Varying NaCl concentrations externally osmotically balanced with glucose (0.1 – 1.1 M) |
| Dynamic network modulation using laser heating | Electroformation  POPC 1 wt.% Rh-PE | DI water  1.5 M sucrose internally  0.75 M NaCl externally |
| Lipid diffusion experiments | Electroformation.  Vesicle 1: POPC, 1 wt.% Rh-PE.  Vesicle 2: POPC | DI water  1.5 M sucrose internally  0.75 M NaCl externally |
| X-ray diffraction of lipid stacks | Bulk hydration of lamellar stacks  POPC 1 wt.% Rh-PE | DI water  Excess hydration (80% DI water) with 0.75 M NaCl |
| Vesicle Communication | Emulsion phase transfer | Buffer: 500 mM KCl, 25mM Tris-HCl, pH 8.0.  Vesicle 1: 1.5 M sucrose, 200 mM CaCl_2,_ 50 ng µl^-1^ α-HL.  Vesicle 2: 1.5 M sucrose, 0.54 mM Fluo-4 1 mM EDTA  10 mM TRIMEB, 0.75 M NaCl externally |
| Membrane Tethers | Anchor localisation experiments: electroformation.  Calcein diffusion experiments: emulsion phase transfer.  POPC 1 wt.% Rh-PE  POPC 1 wt.% NBD-PE  POPC | DI water  Anchor localisation experiments: 1.5 M sucrose internally; 0.75 M NaCl externally  Calcein diffusion experiments: 1.5 M sucrose, 1 mM calcein internally; 0.75 M NaCl externally |
| Vesicle Fusion | Emulsion phase transfer  Vesicle 1: POPC, 1 wt% Rh-PE, 2 wt% 16:0 Biotinyl Cap PE, 150 nm AuNP  Vesicle 2: POPC, 2 wt% 16:0 Biotinyl Cap PE, 150 nm AuNP | DI water  Vesicle 1: 0.5 M sucrose, 50 mM calcein internally  Vesicle 2: 0.5 M sucrose internally  0.25 NaCl externally |
| Cell-free protein expression | Emulsion phase transfer  Vesicle 1: POPC, 2 wt% 16:0 Biotinyl Cap PE, 1 wt.% Cy5-PE, 150 nm AuNP  Vesicle 2: POPC, 2 wt% 16:0 Biotinyl Cap PE, 1 wt,% Rh-PE, 150 nm AuNP  Vesicle 3: POPC, 2 wt% 16:0 Biotinyl Cap PE, 150 nm AuNP | Buffer: PBS, pH 7.4  Vesicle 1: PURExpress solution A in 0.5 M sucrose  Vesicle 2: PURExpress solution B in 0.5 M sucrose, 10 mM magnesium acetate  Vesicle 3: E. coli pJexpress 441 vector for GFP expression in 0.5 M sucorse  0.25 M NaCl externally |

**Supplementary References**

1. Friddin MS*, et al.* Optically assembled droplet interface bilayer (OptiDIB) networks from cell-sized microdroplets. *Soft Matter* **12**, 7731-7734 (2016).

2. Lanigan P*, et al.* Dynamical hologram generation for high speed optical trapping of smart droplet microtools. *Biomedical optics express* **3**, 1609-1619 (2012).

3. Poole C, Losert W. Laser tweezer deformation of giant unilamellar vesicles. *Methods in Membrane Lipids*, 389-404 (2007).

4. Brown AT, Kotar J, Cicuta P. Active rheology of phospholipid vesicles. *Physical Review E* **84**, 021930 (2011).

5. Bendix PM, Oddershede LB. Expanding the optical trapping range of lipid vesicles to the nanoscale. *Nano letters* **11**, 5431-5437 (2011).

6. Li W, Segre P, Gammon R, Sengers J, Lamvik M. Determination of the temperature and concentration dependence of the refractive index of a liquid mixture. *The Journal of chemical physics* **101**, 5058-5069 (1994).

7. Papahadjopoulos D, Nir S, Düzgünes N. Molecular mechanisms of calcium-induced membrane fusion. *Journal of bioenergetics and biomembranes* **22**, 157-179 (1990).

8. Bentz J, Duzgune N, Nir S. Kinetics of divalent cation induced fusion of phosphatidylserine vesicles: correlation between fusogenic capacities and binding affinities. *Biochemistry* **22**, 3320-3330 (1983).

9. Marsh D. *Handbook of lipid bilayers*. CRC Press (2013).

10. Chiruvolu S, Walker S, Israelachvili J, Schmitt F-J, Leckband D, Zasadzinski JA. Higher order self-assembly of vesicles by site-specific binding. *Science*, 1753-1753 (1994).

11. Evans E. Entropy-driven tension in vesicle membranes and unbinding of adherent vesicles. *Langmuir* **7**, 1900-1908 (1991).

12. Koster G, Cacciuto A, Derényi I, Frenkel D, Dogterom M. Force barriers for membrane tube formation. *Physical review letters* **94**, 068101 (2005).

13. Peterman EJ, Gittes F, Schmidt CF. Laser-induced heating in optical traps. *Biophysical journal* **84**, 1308-1316 (2003).

14. Misawa H, Koshioka M, Sasaki K, Kitamura N, Masuhara H. Three‐dimensional optical trapping and laser ablation of a single polymer latex particle in water. *Journal of applied physics* **70**, 3829-3836 (1991).

15. Bailey SM, Chiruvolu S, Israelachvili JN, Zasadzinski JA. Measurements of forces involved in vesicle adhesion using freeze-fracture electron microscopy. *Langmuir* **6**, 1326-1329 (1990).

16. Kučerka N, Liu Y, Chu N, Petrache HI, Tristram-Nagle S, Nagle JF. Structure of fully hydrated fluid phase DMPC and DLPC lipid bilayers using X-ray scattering from oriented multilamellar arrays and from unilamellar vesicles. *Biophysical journal* **88**, 2626-2637 (2005).

17. Jesorka A, Stepanyants N, Zhang H, Ortmen B, Hakonen B, Orwar O. Generation of phospholipid vesicle-nanotube networks and transport of molecules therein. *Nature protocols* **6**, 791-805 (2011).

18. Karlsson M*, et al.* Formation of geometrically complex lipid nanotube-vesicle networks of higher-order topologies. *Proceedings of the National Academy of Sciences* **99**, 11573-11578 (2002).

19. Sott K*, et al.* Controlling enzymatic reactions by geometry in a biomimetic nanoscale network. *Nano letters* **6**, 209-214 (2006).

20. Pautot S, Frisken BJ, Weitz D. Production of unilamellar vesicles using an inverted emulsion. *Langmuir* **19**, 2870-2879 (2003).

21. Fujii S, Matsuura T, Sunami T, Nishikawa T, Kazuta Y, Yomo T. Liposome display for in vitro selection and evolution of membrane proteins. *Nat Protoc* **9**, 1578-1591 (2014).

22. Yamashita D*, et al.* Molecular basis of transmembrane beta-barrel formation of staphylococcal pore-forming toxins. *Nature communications* **5**, 4897 (2014).

23. Gu L-Q, Braha O, Conlan S, Cheley S, Bayley H. Stochastic sensing of organic analytes by a pore-forming protein containing a molecular adapter. *Nature* **398**, 686-690 (1999).

24. Elani Y, Gee A, Law RV, Ces O. Engineering multi-compartment vesicle networks. *Chem Sci* **4**, 3332-3338 (2013).

25. Villar G, Heron AJ, Bayley H. Formation of droplet networks that function in aqueous environments. *Nat Nano* **6**, 803-808 (2011).
