# Supplementary material for: Sculpting and fusing biomimetic vesicle networks using optical tweezers
Source: Nat Commun. 2018 May 14;9:1882. doi: 10.1038/s41467-018-04282-w (PMC5951844; doi:10.1038/s41467-018-04282-w)
Supplement: Supplementary file 2 — Description of Additional Supplementary Files [file 41467_2018_4282_MOESM2_ESM.pdf]

Descriptions of Additional Supplementary Files:

File Name: Supplementary Movie 1
Description: Vesicle adhesion and VIM formation

File Name: Supplementary Movie 2
Description: Constructing and reconfiguring vesicle networks

File Name: Supplementary Movie 3
Description: Formation of tethers

File Name: Supplementary Movie 4
Description: Laser-mediated fusion of gold nanoparticle labelled vesicles
